# Systematic review of economic evaluations in thalassaemia screening programmes globally: developing guidance for low- and middle-income (LMIC) settings

**DOI:** 10.1136/bmjopen-2025-108768

**Published:** 2026-05-06

**Authors:** Katherine Massey, Koukeo Phommasone, Aditi Mehta, Vivienne Qi Yan Lee, Elizabeth A Ashley, Mayfong Mayxay, Chris Painter

**Affiliations:** 1 Costello Medical, Singapore; 2 Lao-Oxford-Mahosot Hospital-Wellcome Trust Research Unit (LOMWRU), Mahosot Hospital, Vientiane, Lao People’s Democratic Republic; 3 Costello Medical, London, UK; 4 Centre for Tropical Medicine and Global Health, Nuffield Department of Medicine, University of Oxford, Oxford, UK; 5 Unit for Health Evidence and Policy (UHEP), Institute of Research and Education Development, University of Health Sciences, Ministry of Health, Vientiane, Lao People’s Democratic Republic; 6 Saw Swee Hock School of Public Health, National University of Singapore, Singapore; 7 Mahidol-Oxford Tropical Medicine Research Unit, Bangkok, Thailand

**Keywords:** Systematic Review, Health policy, Public health, Health economics

## Abstract

**Abstract:**

**Objectives:**

Thalassaemia, a genetic blood disorder, is a major public health burden. Most affected individuals reside in low-and-middle-income countries (LMICs). Screening programmes can reduce incidence, but in resource-constrained settings cost-effectiveness is important. This work aimed to investigate how economic evaluations of thalassaemia screening programmes have been conducted globally, to identify best practices for future evaluation suited to a LMIC context.

**Design:**

Systematic literature review.

**Data sources:**

The original review was undertaken between May and July 2023; an update was completed between November and December 2025. Electronic databases (MEDLINE, Embase, National Health Service Economic Evaluation Database, Health Technology Assessment Database, Cochrane Database of Systematic Reviews), economic databases (Cost-Effectiveness Analysis Registry) and grey literature (including conference proceedings) were searched. Additional validation searches were conducted in Google Scholar to identify relevant studies not indexed in the electronic databases.

**Eligibility criteria:**

Studies were screened against pre-specified criteria by two independent reviewers. Eligible articles reported an economic evaluation of a thalassaemia screening programme for pregnant women or children aged 2 years or younger in any geographic setting.

**Data extraction and synthesis:**

Data extraction for each included article was performed by one author and verified by a second. Findings were interpreted within the context of LMICs, given the high prevalence and resource limitations in these settings. The quality of each article was assessed using the Critical Appraisal Skills Programme Economic Evaluation Checklist; quality assessment for each article was performed by one author and verified by a second.

**Results:**

Of 2112 publications identified from database searches, ten were ultimately included: three cost-effectiveness analyses (CEAs), six cost-benefit analyses (CBAs) and one cost-utility analysis. Study quality varied widely, with most not reporting methodological details such as discounting rates and time horizon. Additionally, no studies employed standard cost-effectiveness metrics, such as quality-adjusted life-years. Seven studies adopted simplified approaches to evaluating thalassaemia screening programmes, relying on basic cost comparisons without formal modelling. Three studies used a decision tree model structure based on the chronological sequence of steps during the screening process. Of these, two Thailand-based studies were notable, given their robust decision tree model, explicit adoption of a lifetime time horizon, application of a discounting rate in line with Thai Health Technology Assessment Guidelines, and performance of sensitivity analyses using recognised methods.

**Conclusions:**

The frequent use of simple cost comparisons likely reflects the complexities surrounding modelling of thalassaemia screening programmes. While traditional CEAs are predominantly used in cost-effectiveness research, practical and ethical challenges associated with calculating health utility differences in this context may limit their use. However, the absence of standard metrics does not preclude a robust economic evaluation, as evidenced by the two high-quality Thailand-based studies. The methods outlined in these papers can be used as a starting point for future economic evaluations, provided the evaluation is further tailored to the local setting. If development of a model is not possible, a simpler CBA with a robust, comprehensive approach could be used. In any case, it is vital to capture the societal benefits of screening programmes; any future evaluation within this context should therefore include a broad societal perspective.

**PROSPERO registration number:**

CRD42023445001.

**STRENGTHS AND LIMITATIONS OF THIS STUDY**

- This systematic literature review (SLR) was conducted in accordance with best-practice methods and guidance from the University of York Centre for Reviews and Dissemination; as such, the review methodology was robust and systematic.
- Manual searches of conference proceedings, economic databases and reference lists were undertaken alongside additional validation searches, contributing to the comprehensive nature of the SLR and ensuring all relevant literature published in the English language was identified.
- This SLR focused on thalassaemia and as such did not capture evaluations of screening programmes for haemoglobinopathies more generally; taking a broader approach may have allowed a larger body of high-quality evaluations to be identified.
- As only literature published in the English language was eligible for inclusion in the SLR, and only English databases were searched, relevant literature published in the native languages of countries that experience significant burden of thalassaemia would not have been identified.

## Introduction

 Thalassaemia is a heterogeneous group of genetic blood disorders characterised by decreased haemoglobin production, resulting in anaemia.^1 2^ The highest incidence and prevalence of thalassaemia is reported in East and South-East Asia, the Mediterranean and the Middle East,^2 3^ with up to 80% of individuals with thalassaemia residing in low- and middle-income countries (LMICs).^1^ Within South-East Asia, Cambodia and Laos have the highest prevalence and mortality rates.^3^ However, it has been challenging to produce robust epidemiological estimates for thalassaemia in this region due to differences in thalassaemia risk between ethnic groups.^4^,^7^

Thalassaemia can present differently according to the affected haemoglobin (Hb) chain (α or β) and disease severity (major, intermediate or minor).^1 8^ Patients with thalassaemia major require lifelong blood transfusions,^2 8^ and if left untreated, the condition can be fatal often before the age of 3 years.^3^ Patients with transfusion-dependent thalassaemia must be carefully managed to limit the number of transfusions required and avoid the negative consequences of iron overload.^9^ All patients with thalassaemia can experience a wide range of other complications which impact quality of life, such as infections, fatigue, musculoskeletal and bone deformities and cardiac conditions.^9^

Given the need for lifelong treatment for affected individuals, LMICs with high thalassaemia prevalence experience a significant healthcare burden associated with thalassaemia, with maintaining adequate blood supplies for transfusions being particularly challenging. In Laos, ~30% of the healthcare system’s overall blood supply is used by individuals with thalassaemia,^10^ and Lao Red Cross has reported struggles with both meeting demand and providing an adequate supply to individuals with thalassaemia.^11^ Given that Laos does not meet WHO’s recommendations for blood donation levels,^11^ the prevalence of thalassaemia puts significant pressure on this limited resource. A similar trend of low blood donation rates is observed throughout South-East Asia and low-income countries report low numbers of voluntary non-remunerated donors.^12^ While blood supplies could be increased by paid donation or family replacement, such schemes would further increase the burden placed on families of patients with thalassaemia or add to the considerable healthcare system cost associated with thalassaemia.^13^

Considering this, and the lack of affordable curative therapies for thalassaemia,^9 14^ reducing incidence through screening programmes may be the most sustainable approach for alleviating the disease burden in LMICs. Screening programmes aim to identify carriers of the disease and/or couples with a high risk of having a child with thalassaemia in order to reduce the number of thalassaemia births. Screening programmes may also incorporate education, family planning information and counselling. Programmes which screen pregnant women may also offer antenatal diagnosis and counselling around continuation or termination of the pregnancy.^15^

Thalassaemia screening programmes, such as Thailand’s nationwide prevention programme, have been shown to reduce the incidence of thalassaemia in countries experiencing significant disease burden.^16^ In addition to the benefits of reducing thalassaemia cases in the short term, as new and potentially curative technologies such as gene therapies (eg, exagamglogene autotemcel) become more widely available, screening programmes may allow for early detection of individuals eligible for treatment. Nonetheless, a number of LMICs with high thalassaemia prevalence, such as Cambodia and Laos, have not established such screening programmes, nor have the capacity to test for carriers of thalassaemia.^16^ However, while screening programmes are likely to promote a more sustainable healthcare system in the long term, it is important to understand whether introducing thalassaemia screening programmes is cost-effective, particularly in resource-limited settings. It is therefore valuable to undertake economic evaluations of such programmes in LMICs, but it is unclear how these evaluations should best be performed.

The objective of this study was to conduct a systematic literature review (SLR) to investigate how economic evaluations of thalassaemia screening programmes have been carried out globally, in order to identify best practices for performing future evaluations in LMIC contexts.

## Methods

The SLR was conducted in compliance with methodological principles of conduct for SLRs, as detailed in the University of York Centre for Reviews and Dissemination’s *“*Guidance for Undertaking Reviews in Health Care*”*, and is reported in line with the Preferred Reporting Item of Systematic Review and Meta-analyses (PRISMA) guidelines.^17 18^ The SLR protocol was registered in the international prospective register of systematic reviews (PROSPERO; CRD42023445001).^19^

The original SLR was undertaken between May and July 2023; an update was subsequently completed between November and December 2025 to capture recent, relevant publications.

### Search strategy

The search strategy was developed using the Population, Intervention, Comparator, Outcome and Study Design framework. Keywords such as ‘thalassaemia’, ‘screening’, ‘economic model’ and ‘economic evaluation’ were included. Searches for the original SLR were conducted in MEDLINE, Embase, National Health Service Economic Evaluation Database (NHS EED), Health Technology Assessment Database (HTAD) and Cochrane Database of Systematic Reviews (CDSR) on 29 May 2023. All electronic databases except NHS EED were searched again on 19 November 2025; NHS EED was not searched for the SLR update as updates to this database ceased at the end of March 2015. For the SLR update, results from the searches were de-duplicated against the results of the original SLR (online supplemental tables 1**–**5).

Proceedings for relevant conferences between May 2021 and December 2025 were searched by hand (online supplemental table 6); searches were restricted to the 2 years prior to the start of the review under the assumption that high-quality conference presentations would subsequently be published as journal articles and captured via database searches.

Supplementary searches of the Cost-Effectiveness Analysis Registry were conducted for any additional relevant studies (online supplemental table 7). Validation searches using a targeted approach were used to identify additional relevant studies published in journals not indexed in the electronic databases. An iterative approach was used in Google Scholar and the first 60 hits of each search were reviewed; these validation searches were repeated on 18 December 2025 using the same protocol (online supplemental table 8). Finally, the bibliographies of relevant SLRs, network meta-analyses, economic evaluations and Health Technology Assessments (HTA) identified through the searches were hand searched to identify any additional studies of relevance.

### Study selection

Studies were eligible for inclusion if they met the eligibility criteria (online supplemental table 9). In brief, included studies were those reporting the outcomes of economic evaluations of screening programmes for thalassaemia for pregnant women or children aged 2 years or younger. Economic evaluations^20^ (cost-utility analyses (CUAs), cost-consequence analyses, cost-effectiveness analyses (CEA), cost-benefit analyses (CBA), cost-minimisation analyses and budget impact analyses) conducted in any country and reporting outcomes including (but not limited to) incremental cost-effectiveness ratios (ICERs), quality-adjusted life years (QALYs) or disability-adjusted life years (DALYs), life years gained and incremental costs were eligible for inclusion. The exclusion criteria included, but were not limited to, studies in animal subjects, studies not presenting relevant economic outcomes and studies reporting screening programmes exclusively for any other disease.

Potentially eligible studies were screened based on title and abstract by two authors independently (KM, KP, VQYL, AM, CP). If necessary, any disagreements were resolved by discussion until a consensus was met, or with the assistance of a third independent reviewer (KM, CP). Final selection of studies was based on full-text reviews, which were undertaken by two authors independently (KM, KP, VQYL, AM, CP); as before, any disagreements were resolved by discussion or, if necessary, with the assistance of a third independent reviewer (KM, CP).

### Data extraction

Relevant data from full-text publications and conference proceedings (abstracts, posters or oral presentations) were extracted into a pre-defined extraction grid in Microsoft Excel. The following data items were considered: screening programme design (including geographic location and duration), details of participants (including number screened, race or ethnicity and gestational period), study design, perspective(s), time horizon, discounting, data source(s), comparator(s), costs measured, screening outcomes and main result (including ICER). Full details on the outcomes and other variables sought for extraction are provided in the online supplemental information.

Data extraction was performed by one author for each included study, after which a second author independently verified the extracted information (KM, KP, VQYL, AM, CP); any discrepancies were arbitrated by a third author (KM, CP).

### Quality assessment

The quality of all included articles was assessed using the Critical Appraisal Skills Programme (CASP) Economic Evaluation Checklist^21^; quality assessment was performed by a single author for each included study and then independently verified by a second author (KM, KP, VQYL, AM, CP). Any discrepancies identified by the second author were arbitrated by a third author (KM, CP). The CASP Economic Evaluation Checklist provides a systematic approach to the critical appraisal of economic evaluations of a broad range of methodologies.

### Patient and public involvement

Patients and the public were not specifically involved in the design, conduct, reporting or dissemination plans of our research. Details regarding researcher reflexivity are provided in the Author Reflexivity Statement, included in the online supplemental file 1.

### Ethics approval

As this was a systematic review of published research, with no use of individual patient data, ethics approval was not required for this work.

## Results

### Identification of selected studies

**Figure 1:**
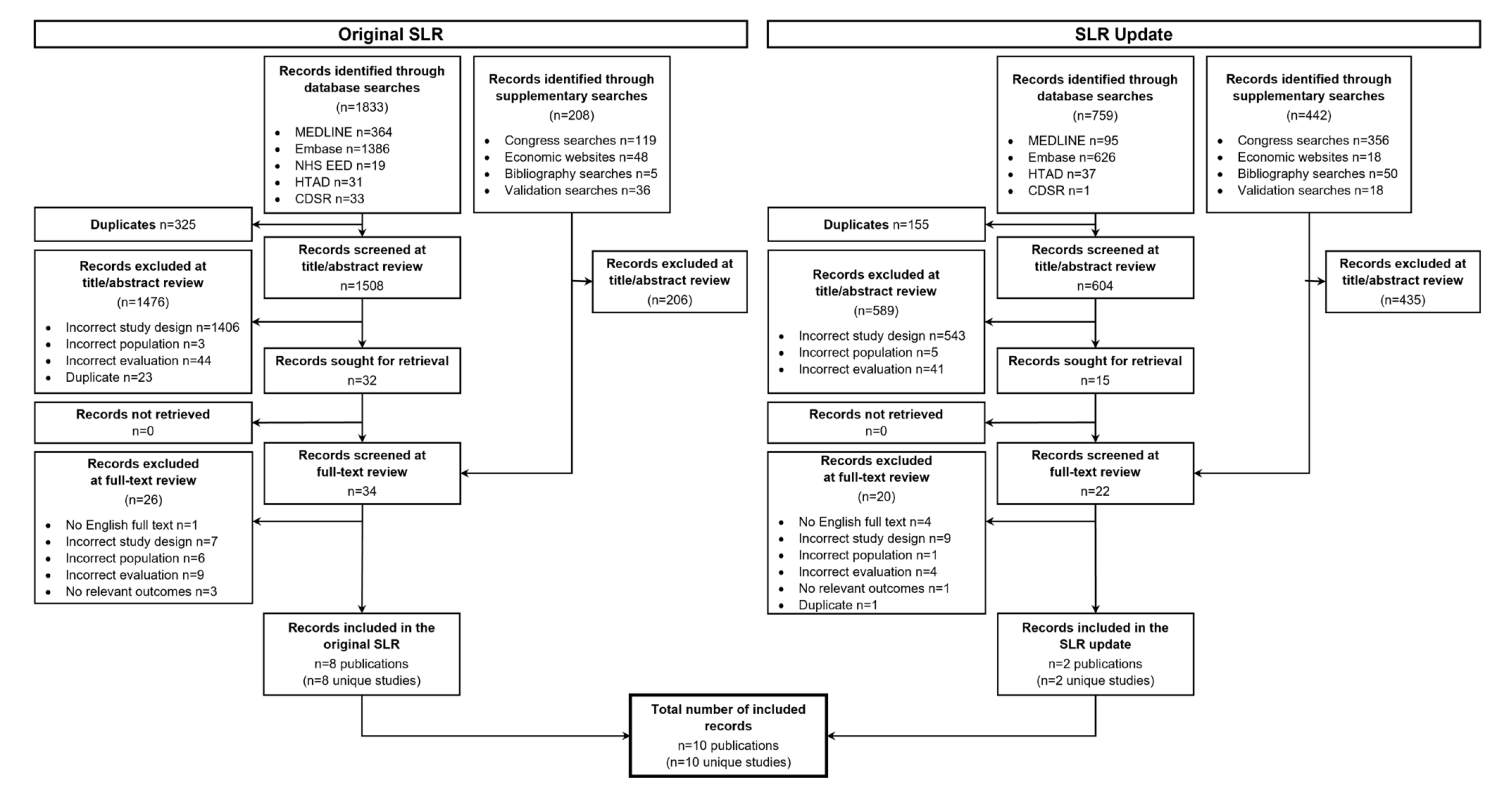
PRISMA flow diagram for economic evaluations identified in the SLR(s). CDSR, Cochrane Database of Systematic Reviews; HTAD, Health Technology Assessment Database; NHS EED: NHS Economic Evaluation Database; SLR, systematic literature review.

The PRISMA flow diagram for the original SLR and SLR update is presented in figure 1. In the original SLR, 1833 records were identified from the database searches. After removing duplicates, 1508 records were screened at the title/abstract review stage. An additional 208 records were identified through supplementary searches. Thirty-four full-texts were reviewed and eight publications reporting eight unique studies were included in the original SLR. In the SLR update, 759 records were identified from the database searches; after the removal of duplicates, 604 were screened at the title/abstract review stage. An additional 442 records were identified through supplementary searches. Twenty-two potentially eligible full texts were reviewed and two publications reporting two unique studies were included in the SLR update. Ultimately, 10 studies were included in this review. Further details for the excluded studies are provided in online supplemental tables 10,11.

The quality of studies varied widely (online supplemental tables 12,13), with most studies not reporting several relevant details on the economic evaluation to demonstrate its robustness. For instance, many studies did not provide a clear description of the study objective or comparators. This decreases the study credibility and could also invite questions about the appropriateness of the study comparator. Furthermore, some studies did not report the time horizon, which is an important component for an evaluation where the lifetime cost of treatment is used to calculate the cost-benefit between the cost per averted case of thalassaemia with a screening programme and the cost per case of thalassaemia. Additionally, discounting was reported in a limited number of studies.

### Study characteristics

Across the included studies, three were published within the last 5 years (2022,^22^ 2025^23 24^) publication year of the other seven studies ranged from 1998 to 2016. All studies evaluated screening programmes targeting pregnant women and evaluations spanned six countries and/or administrative regions: China,^25^ England,^26 27^ Hong Kong,^28^ Israel,^29 30^ Sri Lanka^22^ and Thailand.^23 24 31^

### Screening programmes

Screening programmes ranged in scope from local to national, and all but one were universal.^25^ Seven studies reported screening sites, which included hospitals,^23 24^ primary care,^26^ community clinics,^27 30^ prenatal clinics^28^ and maternal-fetal medicine centres.^25^ Additional methodological details of each screening programme are presented in table 1.

**Table 1:** Methodologies used by screening programmes

Study	Geography	Programme duration	Screening method	Number of patients screened	Gestational period at screening	Follow-on steps
Amarasinghe^22^	Sri Lanka	NR	Full blood count (red cell indices/Hb); HPLC; cation-exchange	280 729	First trimester	Partner screened if woman has β-thalassaemia trait; TOP offered if fetus found to have thalassaemia major
Bryan^26^	England	NR	NR	1454	First trimester	Partner offered screening if woman’s carrier test is positive; at-risk pregnancies offered pre-natal diagnosis; TOP offered if fetus is diagnosed
Cronin^27^	England	NR	Full blood count; isoelectric focusing; HPLC; zinc protoporphyrin assay	2101	NR	Partner screened if pregnant woman has Hb variant, β-thalassaemia traits or possible α-thalassaemia traits; counselling undertaken and pre-natal diagnosis offered
Ginsberg^29^	Israel	NR	Blood count; Hb concentration; RBC count; cell volume; electrophoresis	120 158	NR	Women referred for electrophoresis screening based on abnormal Hb concentration, RBC count and/or low mean cell volume, after which partners receive electrophoresis screening; positive couples receive genetic counselling; TOP offered if fetus is found to have thalassaemia
Koren^30^	Israel	24 years	Blood count; HPLC	~75 000	NR	Partner screened if woman has abnormal screening results; genetic counselling provided to couple at risk of having an affected offspring; TOP recommended if fetus found to be affected
Leung^28^	Hong Kong	5 years	Hb; MCV	18 936	After first trimester	Partner tested if woman’s MCV was <80 fL; couples with suspected/confirmed thalassaemia counselled at the clinic; confirmed cases undergo DNA analysis; CVS and amniocentesis offered to all women at risk of carrying fetus with β-thalassaemia major; serial ultrasound examinations offered as an alternative to CVS and amniocentesis for women at risk of α^0^-thalassaemia; TOP offered if fetus is found to have thalassaemia
Malasai (a)^24^	Thailand	NR	MCV/MCH with DCIP screening; Hb typing test	4062	NR	Partners screened if woman’s initial test (MCV, MCH or DCIP) was positive; if both positive, couple is offered a Hb typing test to confirm carrier status. If Hb typing test is positive, genetic testing for α-thalassaemia, β-thalassaemia or both is offered to confirm carrier status. Women screened without genetic testing proceed directly to final diagnosis via amniocentesis to assess if fetus has thalassaemia
Malasai (b)^23^	Thailand	NR	Strategy 1: MCV/MCH and DCIP in pregnant women; Strategy 2: MCV/MCH with Hb typing in pregnant women; Strategy 3: MCV/MCH with DCIP in both partners	NR	NR	Strategy 1: If a woman tests positive, at-risk partner is offered the same screening to determine carrier status. If both are positive, Hb typing is offered Strategy 2: If a woman tests positive, at-risk partner is offered the same test to determine carrier status Strategy 3: If one partner has a positive screening result, no further testing is performed. If both partners test positive, Hb typing is offered In all strategies, if either partner is suspected of α-thalassaemia or β-thalassaemia carrier status based on Hb typing, DNA analysis is performed to confirm carrier status. For high-risk couples, prenatal diagnostic procedure is offered to confirm fetus’ thalassaemia status. If the fetus is affected by severe thalassaemia, the couple is offered TOP
Wiwanitkit^31^	Thailand	NR	RBC index determination (MCV, MCH); application of mathematical model (parameters: MCV, total RBC count, haemoglobin); DCIP test; Hb electrophoresis	NR	NR	NR
Yang^25^	Mainland China	5 years	Serial ultrasound examination of anaemic markers*; CVS; amniocentesis; cordocentesis; α-genotyping; QF-PCR	1923	11–14 weeks; 15–20 weeks; >20 weeks	Invasive test offered if abnormal ultrasonographic findings were detected

* Authors did not specify which anaemic markers were used.

CVS, chorionic villus sampling; DCIP, dichlorophenolindophenol; DNA, deoxyribonucleic acid; Hb, haemoglobin; HPLC, high-performance liquid chromatography; MCH, mean corpuscular haemoglobin; MCV, mean corpuscular volume; NR, not reported; QF-PCR, quantitative fluorescence-polymerase chain reaction; RBC, red blood cell; TOP, termination of pregnancy.

### Economic evaluations

Across the ten included studies, three presented analyses described as CEAs,^22 23 26^ six presented CBAs^2425 27^,^30^ and one presented an analysis described as a CUA.^31^ All of the included studies comparing thalassaemia screening programmes vs standard of care concluded that implementation of thalassaemia screening programmes was economically favourable.^2225^,^30^ Further details of the results of the economic evaluations are provided in table 2.

**Table 2:** Results of economic evaluations

Study	Type of economic evaluation	Cost year	Description of intervention	Description of comparator	Results	Conclusion
Amarasinghe^22^	Cost-effectiveness	NR	Antenatal screening (diagnosis and termination)	Screening programme for school children, young employees and students	Costs: intervention: USD904 186; comparator: USD104 788 Cost per clinical outcome: intervention: USD20 084; comparator: USD87 324 Number of β-thalassaemia major births prevented per year: 45 Benefit-cost ratios:* intervention: 1.74; comparator: 0.33 Additional cost for preventing a thalassaemia major birth: USD18 159	Implementing an antenatal screening programme would be more beneficial than a screening programme for school children, young employees and students, due to a higher benefit-cost ratio for the antenatal screening programme
Bryan^26^	Cost-effectiveness	2010	Primary care parallel screening (primary care screening with test offered to mother and father together)	Midwife care (sequential screening at the first midwife consultation)	Costs (95% CI): intervention: GBP201 000 (GBP169 000, GBP225 000); comparator: GBP145 000 (GBP119 000, GBP167 000); increment: GBP56 000 Women screened by 70 days (95% CI): intervention: 2556 (1276, 4444); comparator: 264 (92 580); increment: 2292 Cost per additional woman screened by 70 days: GBP25	Both primary care parallel and primary care sequential screening would allow more women to be screened by 70 days than midwife care screening, but at an additional cost. A primary care sequential screening programme would dominate a primary care parallel screening programme on the basis of having a lower cost and higher screening rate
			Primary care sequential screening (primary care screening with test offered to mother and then father only if the mother is a carrier)	Midwife care (sequential screening at the first midwife consultation)	Costs (95% CI): intervention: GBP178 000 (GBP149 000, GBP203 000); comparator: GBP145 000 (GBP119 000, 167 000); increment: GBP33 000 Women screened by 70 days (95% CI): intervention: 2887 (1509, 4930); comparator: 264 (92 580); increment: 2623 Cost per additional woman screened by 70 days: GBP13	
Cronin^27^	Cost-benefit	1994–1995	Antenatal screening programme for haemoglobinopathies	No screening (lifetime care costs)	Costs: intervention: GBP42 629; comparator: GBP6113 Number of pregnancies at risk identified and number of sickle cell or thalassaemia births: 12 Savings in health service costs per case of β-thalassaemia averted: GBP123 000	An antenatal screening programme would be economically beneficial compared with no screening programme, resulting in savings in health service costs
Ginsberg^29^	Cost-benefit	1996	Prenatal screening	No screening (lifetime care costs)	*Healthcare sector perspective* Costs: intervention: USD900 197 Cost per clinical outcome: intervention: USD67 369; comparator: USD284 154 Number of cases of thalassaemia averted: 13.4 Benefit-cost ratio†: 4.22/1	A screening programme would be beneficial as a result of the number of thalassaemia cases that could be averted and the lifetime costs associated with caring for a person with thalassaemia
					*Societal perspective* Costs: intervention: USD900 197; Cost per clinical outcome: intervention: USD67 369 Number of cases of thalassaemia averted: 13.4 Benefit-cost ratio†: 6.01/1	
Koren^30^	Cost-benefit	2011	Prenatal screening (diagnosis and termination)	No screening (lifetime care costs)	Costs: intervention: USD413 795 Cost per clinical outcome: intervention: USD63 660; comparator: USD1 971 380 Number of cases of thalassaemia averted: 110 Net saving (prevention of 45 affected newborns over a 10-year period, after deducting the cost of operating the prevention programme): USD60 000 000	Implementing a screening programme in this setting would result in net savings arising from the treatment costs averted as a result of fewer thalassaemia births
Leung^28^	Cost-benefit	2001–2002	Prenatal screening	No screening (lifetime care costs)	Costs: intervention: HKD10 020 686 Cost per clinical outcome: intervention: HKD14 567; comparator: HKD2 745 600 Number of β-thalassaemia births averted: 18 Net saving (prevention of 18 affected newborns, after deducting the cost of operating the prevention programme): HKD40 400 000	Implementing a screening programme in this setting would result in cost savings due to averting the lifetime care costs associated with thalassaemia births
Malasai (a)^24^	Cost-benefit	2023	Prenatal screening and genetic testing	Prenatal screening only	Costs: intervention: USD427.73; comparator: USD427.76 Cost avoidance due to averted α- and β-thalassaemia births: intervention: USD1 995.48; comparator: USD1505.54 Net benefit: USD490	Implementing a standard thalassaemia screening programme with genetic testing results in an observed cost saving compared with screening without genetic testing
Malasai (b)^23^	Cost-effectiveness	2024	Strategy 2 (prenatal screening using MCV/MCH and Hb typing)	Strategy 1 (prenatal screening using MCV/MCH and DCIP)	Costs: intervention: USD46.15; comparator: USD21.95; increment: USD24.2 Number of cases averted: intervention: 0.00657; comparator: 0.00056; increment: 0.00602 Cost per case averted: USD4023	Relative to Strategy 1, Strategies 2 and 3 were found to be cost-effective; Strategy 3 was the most cost-effective approach
			Strategy 2 (prenatal screening using MCV/MCH and Hb typing)	Strategy 3 (couple screening using MCV/MCH and DCIP)	Costs: intervention: USD46.15; comparator: USD35.44; increment: USD10.71 Number of cases averted: intervention: 0.00657; comparator: 0.00541; increment: 0.00116 Cost per case averted: USD9218	
			Strategy 3 (couple screening using MCV/MCH and DCIP)	Strategy 1 (prenatal screening using MCV/MCH and DCIP)	Costs: intervention: USD35.44; comparator: USD21.95; increment: USD13.49 Number of cases averted: intervention: 0.00541; comparator: 0.00056; increment: 0.00485 Cost per case averted: USD2779	
Wiwanitkit^31^	Cost-utility	NR	RBC index determination	Hb electrophoresis screening method	Cost/utility‡ rate (RBC index determination): 106.1	Compared with Hb electrophoresis, the screening method with the lowest cost per accurate diagnosis was DCIP
			Application of mathematical model		Cost/utility‡ rate (application of mathematical model): 81.9	
			DCIP		Cost/utility‡ rate (DCIP): 51	
			Hb electrophoresis		Cost/utility‡ rate (Hb electrophoresis): 200	
Yang^25^	Cost-benefit	NR	Non-invasive prenatal diagnosis programme (ultrasound)	Conventional invasive prenatal diagnosis tests	Costs: intervention: USD213 383; comparator: USD554 810; increment: –USD356 499 Benefit: Reduction in invasive testing by 75% without missing cases of homozygous α-thalassaemia and associated reduction in spontaneous abortion Savings per patient with an unaffected pregnancy: USD246 Number of spontaneous abortions associated with invasive procedures prevented: 15	A non-invasive prenatal diagnosis programme would be beneficial compared with conventional invasive prenatal testing due to lower programme costs

* Benefit-cost ratio=benefits of programme (average lifetime costs of treatment per patient * number of births prevented)/costs of programme

† Benefit-cost ratio=benefits of programme (average lifetime cost of treating homozygotes * number of homozygote births prevented)/costs of programme (costs of educational programme+costs of screening)

‡ Utility was defined as each method’s rate of ability to detect an accurate result (number of abnormal cases/number of true cases).

DCIP, dichlorophenolindophenol; GBP, Great Britain Pound (British pound sterling); Hb, haemoglobin; HKD, Hong Kong dollar; MCH, mean corpuscular haemoglobin; MCV, mean corpuscular volume; NR, not reported; RBC, red blood cell; USD, United States dollar.

### Approaches

The majority of studies (seven out of ten) adopted simplified approaches to evaluating thalassaemia screening programmes, relying on basic cost comparisons without formal modelling. For example, Amarasinghe *et al* compared the costs and benefits associated with three new screening strategies, one of which was antenatal screening, vs the current screening programme.^22^ An ICER, expressed as the additional cost required to prevent a thalassaemia major birth, was calculated by dividing the difference in total costs associated with each screening strategy by the difference in number of births averted.^22^ Four studies performed direct cost comparisons between antenatal screening programmes and no screening or standard of care, specifically evaluating how lifetime treatment costs associated with a thalassaemia patient compared with the cost of preventing one affected newborn through screening.^27^,^30^ Yang *et al* took a similar approach to these four studies, but evaluated the costs associated with a non-invasive prenatal diagnosis programme relative to the costs associated with an invasive diagnostic approach, to estimate the cost savings associated with avoiding invasive testing in unaffected pregnancies.^25^ Wiwanitkit did not consider costs of a screening programme at all, and instead compared the costs associated with four different screening modalities against their diagnostic performance.^31^

Three studies identified in the review used a decision tree model structure based on the chronological sequence of steps during the screening process.^23 24 26^ These steps were similar across all three studies, spanning initial screening of pregnant women, subsequent partner screening and pregnancy outcomes (including termination of pregnancy). Malasai *et al* (a) and Malasai *et al* (b) analysed screening programmes targeted towards pregnant women of Thai nationality.^23 24^ Malasai *et al* (a)^24^ compared the costs and benefits of a standard thalassaemia screening programme with or without genetic testing; the benefits were considered to be the cost avoidance achieved by averting cases of thalassaemia and productivity gains for caregivers not required to care for children with the condition. Malasai *et al* (b)^23^ evaluated the costs and outcomes associated with three different screening algorithms, using different orders of screening tests and populations: (1) all pregnant women are screened using mean corpuscular volume (MCV)/mean corpuscular haemoglobin (MCH) and dichlorophenolindophenol (DCIP), (2) all pregnant women are screened using MCV/MCH and Hb typing and (3) both partners are screened using MCV/MCH and DCIP. Malasai *et al* (b)^23^ reported ICERs as the primary economic outcome, defined as the cost per severe thalassaemia case prevented. While the specific methodology and outcomes differed between these two papers, the decision tree approaches utilised were extremely similar: both models incorporated transitional probability parameters, including the probability of testing positive for each screening test, acceptance rates, the prevalence of severe thalassaemia and the rate of pregnancy termination.^23 24^

Bryan *et al*, the third study to employ a decision tree model structure, compared the costs and outcomes associated with three different screening approaches: (1) primary care parallel (screening offered to both partners at the same time), (2) sequential screening (screening offered to the pregnant woman first and to the partner only if the woman received a positive result) at the first midwife consultation and (3) primary care sequential screening.^26^ While the decision tree model developed by Bryan *et al* included parameters similar to the Malasai *et al* (a) and Malasai *et al* (b) studies, the authors adopted a broader scope, considering additional factors such as the declared father not being the biological father and the fetus failing to reach term.^23 24 26^ Bryan *et al* also included the probability of inter-ethnic unions, likely due to the fact that the research was based in the UK, where the general population is multiethnic; consequently, the prevalence of carrier status varies and must be taken into account.^26^ Similar to Malasai *et al* (b)^23^, Bryan *et al* reported ICERs as the primary economic outcome, although the definition differed: cost per additional woman screened before 70 days of gestation.^26^

Thus, none of the included studies used metrics such as QALYs or DALYs in their analyses. Where studies reported their outcomes in the form of ‘ICERs’, these were generally calculated as costs per benefit, such as thalassaemia births avoided.

### Perspectives

Five studies explicitly reported the perspective taken by their evaluation.^22^,^2426 29^ Of these, Amarasinghe *et al* and Bryan *et al* took only a healthcare sector perspective; specifically, the perspective of the Ministry of Health Sri Lanka and UK National Health Service, respectively.^22 26^ Conversely, Ginsberg *et al* adopted both a healthcare sector (Ministry of Health Israel) and societal perspective. Malasai *et al* (a) and Malasai *et al* (b) took a societal perspective.^23 24^ The remaining five studies did not explicitly report a perspective, but instead reported their evaluations from a specific context, such as a specific department (eg, a UK haematology department) or healthcare centre (eg, a mainland Chinese medical centre).^25 27 28 30 31^

### Time horizons

Four studies explicitly reported the time horizon over which the analysis was conducted.^22^,^2426^ Amarasinghe *et al* implemented a 10-year time horizon, due to the Ministry of Health having a 5–8 year time frame for implementation of the screening programme.^22^ Bryan *et al* modelled a time horizon of <1 year, which covered the duration of pregnancy to conclusion (ie, birth, termination or other pregnancy loss unrelated to screening).^26^ Malasai *et al* (a) and Malasai *et al* (b) both modelled costs and benefits throughout pregnancy and the lifetime of individuals with thalassaemia.^23 24^ Two other studies collected data over specific periods of time, and extrapolated these data to calculate lifetime costs/benefits: Leung *et al* and Koren *et al* assessed data from a 4-year and 24-year period, respectively.^28 30^

### Discounting rates

Discounting was explicitly reported in five studies^23 24 26 27 29^; one study deemed discounting to be not necessary, given the short-term horizon of the clinical events.^26^ Conversely, Ginsberg *et al* opted to use a 5% annual discount rate, although no reasoning was provided.^29^ Cronin *et al* adopted a 6% discount rate, as recommended by His Majesty’s Treasury.^27^ Malasai *et al* (a) and Malasai *et al* (b) both applied a 3% discount rate to both costs and outcomes; Malasai *et al* (a) stated this approach aligned with Thai HTA Guidelines.^23 24^ Notably, no other studies carried out in non-high-income countries (HICs) (China, Sri Lanka and Thailand) reported discounting.^22 25 31^

### Sensitivity analyses

Five of the included studies conducted sensitivity analyses; of these, two studies conducted a limited number of deterministic analyses to investigate the impact of specific parameters.^27 29^ Cronin *et al* conducted analyses to evaluate the impact of the proportion of pregnant women choosing to terminate their pregnancy to assess potential overestimation of treatment costs.^27^ Ginsberg *et al* examined the effect of changing the applied discount rate.^29^ The remaining three studies employed standard methods to conduct comprehensive sensitivity analyses: Bryan *et al* performed a probabilistic sensitivity analysis only, whereas Malasai *et al* (a) and Malasai *et al* (b) used both deterministic and probabilistic approaches to investigate key drivers of model results.^23 24^

### Costs

Key details around the calculations of costs were reported inconsistently across studies. For example, three studies did not report the year for which costs were reported, and the types of costs included varied widely among them.^22 25 31^ Specifically, due to the nature of the methodology, Wiwanitkit solely considered the cost per diagnostic test,^31^ while Yang *et al* limited their analysis to the costs of screening and testing only. Conversely, the costs considered in Amarasinghe *et al* employed a more comprehensive approach, including costs associated with fetal sampling and diagnosis as well as cost of transport, consumables, staff salaries, educational materials and medical termination.

The other seven studies, which had reported cost years ranging from 1994 to 2024, all considered direct costs of the programme associated with screening and testing, including prenatal diagnosis and termination of pregnancy.^2324 26^,^30^ Of these, Bryan *et al,* Malasai *et al* (a) and Malasai *et al* (b) were unique in that they considered direct non-medical costs to participants such as travel, food, opportunity costs of pregnant women and caregivers during screening and informal care.^23 24 26^ Four studies accounted for direct costs associated with lifetime care for individuals with thalassaemia,^2228^,^30^ but only two detailed both the standard of care and the associated costs.^29 30^ For Ginsberg *et al* these costs included home infusion service, hospital operations, tests and visits, as well as lost earnings and premature mortality costs^29^; for Koren *et al* these costs included diagnostic workup as well as annual blood transfusions, iron chelator treatment and procedures (such as MRIs and splenectomies).^30^

The inclusion of indirect costs was limited to studies adopting a societal perspective. Ginsberg *et al* considered these in the form of lost earnings and premature mortality for individuals with thalassaemia, while both Malasai *et al* (a) and Malasai *et al* (b) included productivity losses due to illness for individuals with thalassaemia.^23^ ^24^ ^29^

### Assumptions

The majority of studies (seven of ten) reported key assumptions of the analysis,^22^,^2426 27 29 30^ such as life expectancy of patients with thalassaemia.^22 24 27 29 30^ Life expectancy across the studies for patients with β-thalassaemia ranged from 13.5 to 50 years of age,^22 24 27 29 30^ and this assumption was influential in studies calculating lifetime treatment costs. There was a correlation between life expectancy and year of publication, with more recent publications reporting higher life expectancies.^22 24 29 30^ The lowest life expectancy reported (13.5 years in patients with β-thalassaemia major) was from the lowest income country (Sri Lanka).^22^ The highest life expectancy (50 years of age in patients with β-thalassaemia) was from a HIC (Israel).^30^ Only Malasai *et al* (a)^24^ reported an assumed life expectancy for severe α-thalassaemia, likely due to the severity of the condition; this was assumed to be 1 year.

In two studies, authors had to estimate the number of thalassaemia cases prevented due to a lack of empirical data.^22 23^ Other key assumptions made across studies involved working age, and rates of uptake, incidence and annual income growth.^23 24 26 29 30^

## Discussion

Thalassaemia is associated with a significant clinical, humanistic and economic burden of disease, particularly in LMICs across South-East Asia, the Mediterranean and the Middle East where it is highly prevalent.^2 3^ However, many of these resource-limited countries have not implemented programmes to reduce incidence of the disease. This SLR aimed to investigate how economic evaluations, across all geographies, have been previously carried out for thalassaemia screening programmes to understand how future evaluations should be conducted in a LMIC context. To our knowledge, this is the first SLR to investigate how economic evaluations of thalassaemia screening programmes have historically been performed.

This SLR identified a small number of studies that varied widely in quality. The represented geographies varied from countries in which thalassaemia is prevalent (Thailand and Sri Lanka) to those in which thalassaemia is largely limited to ethnic minorities (England). Only five of ten studies were carried out in non-HICs (China, Sri Lanka and Thailand), as defined by the World Bank classification. It was additionally found that the methodologies employed in the studies were diverse, a conclusion also reached by Rahim and colleagues in their SLR exploring whether thalassaemia screening programmes can reduce incidence rates and economic burden.^32^

CBAs evaluate the financial feasibility of an intervention through the monetary comparison between the total costs associated with or without a particular course of action.^33^ CEAs, on the other hand, focus on achieving a specific outcome at the lowest possible cost.^33^ Instead of comparing total costs and benefits, a CEA measures the efficiency of different interventions by calculating the cost per unit of a desired outcome.^33^ The majority of studies identified in this SLR were CBAs of moderate quality and robustness. Only three economic evaluations presented analyses which were described as CEAs; however, they did not use metrics such as QALYs or DALYs, or report traditional ICERs.^22^,^2426^ This finding is contrary to previous systematic reviews of economic evaluations of screening programmes for other conditions, including cervical cancer,^34 35^ cardiometabolic diseases^36^ and hepatitis C,^37^ in which the majority of included studies were CEAs and CUAs.

Nevertheless, the included studies offer useful learnings for conducting economic evaluations of thalassaemia screening programmes in LMIC contexts. While most of the included studies relied on simplified cost comparisons, often not reporting expected methodological details such as discounting rates, perspective and time horizon, the Malasai *et al* (a) and Malasai *et al* (b) studies were notable exceptions.^23 24^ Indeed, the objective of Malasai *et al* (b)^23^ was to provide evidence that could inform policy decisions and guide resource allocation. These studies, which were based in Thailand, demonstrate that robust economic evaluations are feasible in this context. Both studies used a decision tree model structure that incorporated key parameters such as rates of incidence, uptake and prevalence. Furthermore, the authors explicitly adopted a societal perspective, considering costs accordingly and comprehensively, as well as used an appropriate lifetime time horizon and applied a discounting rate of 3%, in line with Thai HTA Guidelines. The two studies also conducted both deterministic and probabilistic sensitivity analyses using recognised methods, resulting in robust conclusions. Thus, while these studies may not align with HTA-level economic evaluations, they can serve as practical blueprints for researchers based in similar contexts.

To note, it is important to consider the rationale behind adopting a particular evaluation approach. Brent and colleagues argue that CBAs are a more relevant economic evaluation method when adopting a societal perspective.^33^ Nonetheless, CEAs may still be the preferred method for conducting economic evaluations due to their ability to standardise comparisons across different health interventions; when employing a generic measure of health status, such as QALYs, CEAs allow for direct comparisons across different disease areas and programmes, facilitating contextualisation of results within the broader healthcare sector.^20^ In contrast, for CBAs there exists no universally accepted monetary value for specific health benefits, leading to variability in approaches and thus challenges when comparing outputs between different CBAs.^20^ Consequently, CEAs have traditionally been the method employed for determining the most cost-effective use of resources in HTAs, as they may support the decision-making process for healthcare policymakers to a greater degree than CBAs.^20^ However, should the decision-making process fall to a policymaker outside of the healthcare sector, CBAs might be more pertinent due to the versatility they offer.^20^ Another consideration is the granularity of data required; for CEAs, robust data are generally necessary to provide accurate estimates. As such, the use of pragmatic CBAs that do not quantify health burden, but rather assign monetary values for both costs and benefits, may indicate complexities around modelling thalassaemia screening programmes; in such cases, modellers may have to trade off the value of calculating cost-effectiveness metrics with ensuring a robust analysis can be performed with the available data. The identified articles exemplify this; for example, Malasai *et al* (b)^23^ conducted a robust CEA but did not report any standard cost-effectiveness metrics.

**Figure 2:**
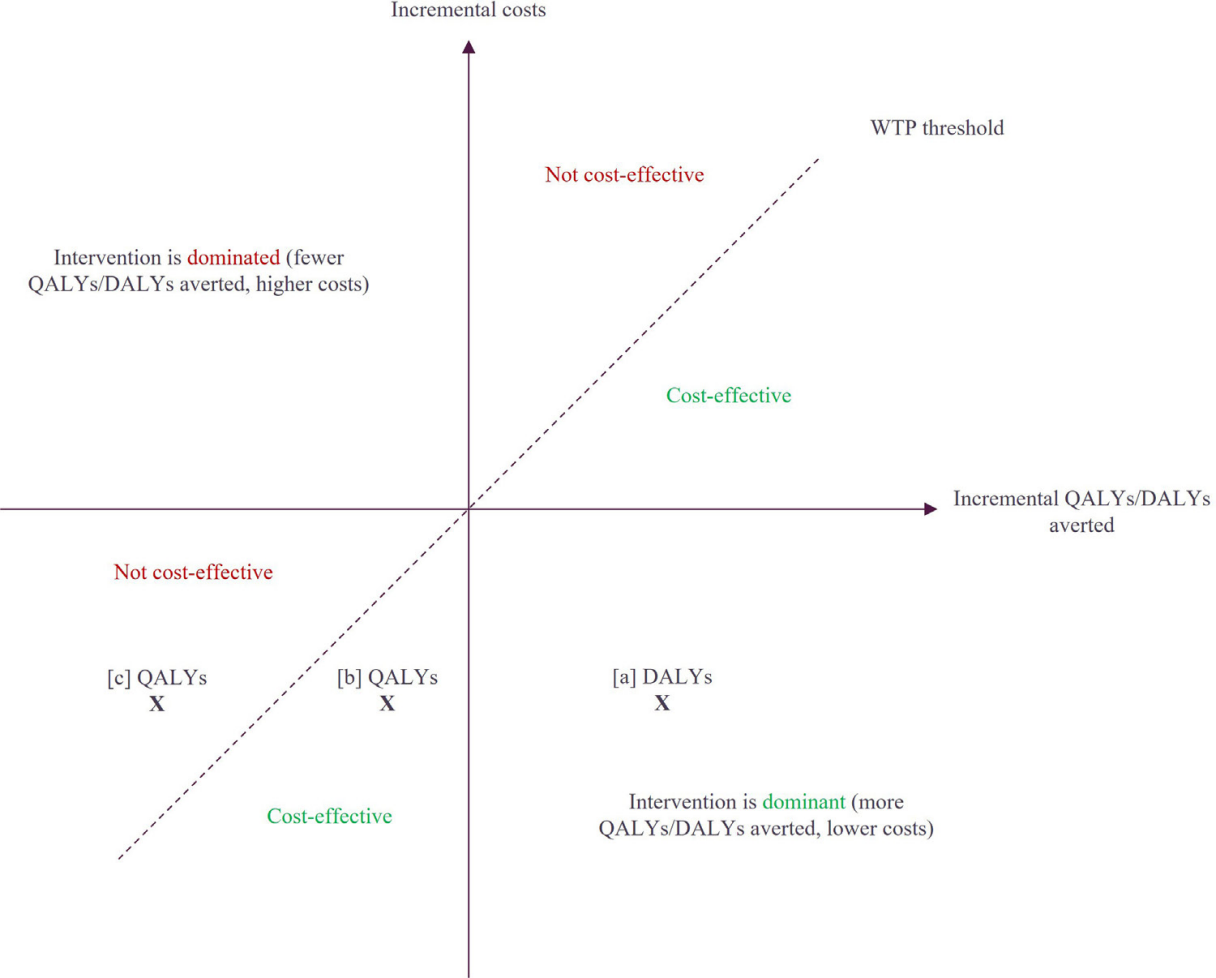
Cost-effectiveness plane displaying potential variation in cost-effectiveness results depending on the choice of QALYs or DALYs. DALY, disability-adjusted life year; QALY, quality-adjusted life year; WTP, willingness-to-pay.

Further, Abel and Quaife as well as Goldhaber-Fiebert and Brandeau report that CEA investigating topics such as fertility, childbearing, contraception and pregnancy raise multifaceted ethical challenges and paradoxes.^38 39^ In this context, when evaluating a thalassaemia screening programme, QALYs of a pregnant woman carrying a fetus diagnosed with thalassaemia who continues with the pregnancy would be directly compared with QALYs of a pregnant woman who terminates the pregnancy. Alternatively, QALYs of an individual with thalassaemia would need to be compared with QALYs of an unborn fetus. Both scenarios raise ethical challenges, as they necessitate considering a hypothetical situation in which a person with a chronic disease is not born. Furthermore, such comparisons could be interpreted as the ‘valuation’ of the life of a person with a chronic disease, particularly when QALYs are used to determine cost-effectiveness. As Goldhaber-Fiebert and Brandeau report, such considerations may be why economic evaluations of health interventions impacting fertility and childbearing often avoid the challenges of assigning QALYs for pregnancy and fertility-related outcomes by instead reporting costs per intermediate outcome.^39^ With this considered, the absence of cost-effectiveness metrics in the studies included in this SLR may be a byproduct of the intricacies around calculating health utility differences in the context of the birth of children with a certain condition vs termination of pregnancy. Nonetheless, even if standard cost-effectiveness metrics are employed when considering prospective child outcomes, modellers must carefully consider whether to use DALYs or QALYs or both where possible, to avoid potential bias. This is because the traditional relationship between QALYs and DALYs no longer holds in such a scenario. For example, if an affected pregnancy is terminated, the potential DALYs averted could be substantial while zero QALYs would be accrued. Figure 2 demonstrates the impact of differing economic evaluation designs: choosing DALYs in [a] could lead to conflicting cost-effectiveness conclusions depending on the size of the QALY differential at points [b] and [c].

These considerations help explain why reporting QALYs may not be feasible in this context, a conclusion further supported by a recent HTA report on carrier screening programmes for haemoglobinopathies and thalassaemia, among other conditions, published by Ontario Health.^40^ Similar to the ICERs captured in this SLR, the HTA report expressed these as additional cost per affected birth avoided; the challenges with estimating QALYs for genetic diagnostic technologies were acknowledged and named as a reason why changes in QALY were not a primary outcome for the cost-effectiveness evaluation undertaken. Overall, this indicates that the absence of QALYs does not preclude a robust economic evaluation and supports the use of the methods outlined in Malasai *et al* (a) and Malasai *et al* (b) as a sensible starting point for future economic evaluations.^23 24^ This is because these studies are based in an LMIC context, include a clear study objective, provide a description and rationale for the competing alternative, perform comprehensive sensitivity analyses, incorporate discounting and specify a time horizon. Importantly, both studies adopt a societal perspective. The economic burden of thalassaemia on patients and their families can be extremely high due to out-of-pocket expenses such as transport to healthcare facilities, costs associated with blood transfusions (eg, if there is a need to obtain the blood privately or if there are charges to the patient at public hospitals) and chelation therapy, and lost wages due to absenteeism.^12 13^ Given the economic burden that many families face as a result of thalassaemia in resource-limited settings without universal health coverage, it is extremely important to understand what the impacts of reducing the prevalence of thalassaemia through the use of a screening programme would have at a societal level. As such, any future economic evaluation within this context should aim to include comprehensive societal cost inputs (ie, both direct and indirect costs), as has been done in these studies.

To note, all future economic evaluations must still be tailored to their specific context. For example, discount rates should align with local or national standards to ensure accurate reflection of the time value of money. Additionally, employing a lifetime time horizon that accurately accounts for average life expectancy of a person living with thalassaemia in the geography of interest would also help ensure a comprehensive evaluation is undertaken. Separately, the variance in the heterogeneity in prevalence of thalassaemia across countries could be taken into account.^4^,^6^ For example, scenario analyses could be conducted for regions or provinces as the cost-effectiveness of screening may vary accordingly. These types of scenario analyses could help LMICs target particularly burdened areas, thereby helping maximise the impact of the screening programmes and ensure that limited resources are used most effectively.

In the event that development of a model is not possible, for example in a context with severely limited resources or a not-yet-established screening programme, a CBA with a straightforward approach (eg, comparing the cost per averted case of thalassaemia vs the cost of lifetime care per case of thalassaemia) could be used. While the robustness and credibility of results should still be maximised as far as possible, such an approach would have less significant requirements for data than a detailed model.

### Strengths and limitations of this study

This SLR was conducted in accordance with best-practice methods and guidance from the University of York CRD. As such, the review methodology was robust and systematic, utilising a pre-specified protocol, search strategy and inclusion/exclusion criteria to identify relevant research. Furthermore, manual searches of conference proceedings, economic databases and reference lists were undertaken alongside additional validation searches, contributing to the comprehensive nature of the SLR and ensuring all relevant literature published in the English language was identified.

However, it is also important to note the limitations of this research. A small number of studies were identified, most of which were published prior to 2015 and only five of which were carried out in non-HICs. Further, the review focused on thalassaemia and did not capture evaluations of screening programmes for haemoglobinopathies more generally; taking a broader approach may have allowed a greater body of literature to be identified and for more conclusions to be drawn with regards to best practice for economic evaluations. Moreover, only publications written in English were included in the review, and no non-English databases, like Global Index Medicus, were searched; as such, relevant literature published in the languages of countries which experience significant burden of thalassaemia would not have been identified. Lastly, it is important to highlight that this SLR only captured evaluations of antenatal screening programmes; no study identified in this review evaluated post-natal screening programmes. However, should potentially curative gene therapy become more accessible in LMICs, economic evaluations of post-natal screening programmes may become more prevalent, and therefore may be an important alternative to consider.

### Policy implications

The findings of this review can be used to inform best practices for conducting economic evaluations of thalassaemia screening programmes in LMICs in the future. The outputs from the resulting economic evaluations can facilitate implementation of thalassaemia screening programmes in resource-poor settings, leading to better management of thalassaemia and, consequently, alleviating the disease burden in the long term.

### Future research

Some of the outstanding questions of this review could be addressed by conducting a broader review of screening for haemoglobinopathies. In particular, such a review may be able to offer further insights with regards to the ethical and technical considerations around calculating utilities, QALYs and ICERs for screening programmes in LMICs, in the context of programmes which aim to prevent the birth of affected individuals. In addition, while comprehensive CEAs were captured in this review, a broader review may supplement these findings and confirm whether comprehensive CEAs, such as those which might be conducted for HTAs, would be feasible or desirable to conduct for a haemoglobinopathy screening programme in a LMIC. Finally, capturing a greater body of literature published in the last ~10 years may provide additional insight into what is currently considered best practice for conducting an economic evaluation of a haemoglobinopathy screening programme in a LMIC. As an accompaniment to such an economic review, a clinical review of screening for haemoglobinopathies could also be undertaken to identify best practices in screening practices across different contexts, including LMIC settings, and intervention design.

## Conclusion

While the dearth of relevant high-quality literature on economic evaluations of thalassaemia screening programmes identified during this SLR make it challenging to interpret the findings, the studies identified provide useful insights into performing an economic evaluation. Conducting and publishing a robust economic evaluation of an existing or hypothetical screening programme in a LMIC setting with a high prevalence of thalassaemia would be a valuable contribution to the existing body of literature. Furthermore, this research suggests that an economic evaluation aligned with the methods employed by Malasai *et al* (a) and Malasai *et al* (b) and tailored to the local setting, would be the preferred approach to evaluating screening programmes in this context.^23 24^ In the event that development of a model is not possible, a simpler CBA conducted using a robust and comprehensive approach would be a pragmatic alternative.

## Supplementary material

10.1136/bmjopen-2025-108768 online supplemental table 1

10.1136/bmjopen-2025-108768 online supplemental file 1

## Data Availability

Data sharing not applicable as no datasets generated and/or analysed for this study.
