# Supplementary material for: Systematic review of economic evaluations in thalassaemia screening programmes globally: developing guidance for low- and middle-income (LMIC) settings
Source: BMJ Open. 2026 May 6;16(5):e108768. doi: 10.1136/bmjopen-2025-108768 (PMC13150896; doi:10.1136/bmjopen-2025-108768)
Supplement: online supplemental table 1 [file bmjopen-16-5-s001.docx]

**Systematic review of economic evaluations in thalassaemia screening programmes globally: developing guidance for low- and middle-income (LMIC) settings**

Dr Katherine Massey;^1^ Dr Koukeo Phommasone;^2^ Aditi Mehta;^3^ Vivienne Lee;^1^ Elizabeth A Ashley;^2,6^ Mayfong Mayxay;^2,4,6,7^ Chris Painter^2,5,6^

*^1^Costello Medical, Singapore*

*^2^Lao-Oxford-Mahosot Hospital-Wellcome Trust Research Unit (LOMWRU), Mahosot Hospital, Vientiane, Lao PDR*

*^3^Costello Medical, London, UK*

*^4^Unit for Health Evidence and Policy (UHEP), Institute of Research and Education Development, University of Health Sciences, Ministry of Health, Vientiane, Lao PDR*

*^5^Mahidol-Oxford Tropical Medicine Research Unit, Bangkok, Thailand*

*^6^Centre for Tropical Medicine and Global Health, Nuffield Department of Medicine, University of Oxford, Oxford, UK*

*^7^Saw Swee Hock School of Public Health, National University of Singapore, Singapore*

**Correspondence to:** Chris Painter (christopher.painter@ndm.ox.ac.uk)

**Key words:** thalassaemia, economic evaluation, systematic literature review, disease prevention

# SUPPLEMENTARY MATERIALS

Supplementary Information: Outcomes and Other Variables Sought for Extraction

Screening Programme and Participant Characteristics

- Screening programme design including the geographical location, screening site, programme scope (e.g. national or regional, universal or targeted to at-risk groups) screening method, type of thalassaemia screened for, details of any partner screening and follow-on steps
- Screening programme duration and duration of any follow-up
- Participant cohort, including the number of participants screened, age, sex, race or ethnicity, gestational period

Economic Outputs

- Evaluation perspective (e.g. societal, healthcare sector etc.)
- Details of the economic evaluation including the type of evaluation (i.e., CEA, CBA, CUA), cost year, type of model, time horizon, cycle length, rationale for study design, discounting, rationale for discounting, details of any treatment sequencing, deterministic/probabilistic sensitivity analyses
- Sources of data used in the analysis for costs, resource use, benefits/outcomes, epidemiology etc.
- Details of the interventions and comparators and any key assumptions made in the evaluations
- Cost outcomes, including intervention and comparator total costs, intervention and comparator costs per clinical outcomes, incremental costs
- Screening outcomes, including description of outcome (e.g. number of women screened, number of cases detected), intervention outcome, comparator outcome, incremental outcome
- Cost-effectiveness outcomes, including ICER and WTP threshold
- Any other economic outcomes including cost-benefit ratio, cost-utility rate

Supplementary Table 1. Search terms for MEDLINE (searched via Ovid SP)

|  |  |  | **Original** | **Update** |
| --- | --- | --- | --- | --- |
|  |  |  | **Date searched: 29th May 2023**  **Records retrieved: 364** | **Date searched: 19th November 2025**  **Records retrieved: 95** |
| **Term group** | # | Search terms | Results | Results |
| **Thalassaemia Screening** | 1 | exp thalassemia/ | 24798 | 26088 |
|  | 2 | (thalass?emia or lepore or hydrops fetalis).ti,ab,kf. | 25816 | 28396 |
|  | 3 | ((Mediterranean or erythroblastic or cooley$) adj an?emi$).ti,kf. | 424 | 424 |
|  | 4 | (h?emoglobin adj6 (disorder$ or defect$ or disease$ or trait$)).ti,ab,kf. | 6567 | 7483 |
|  | 5 | or/1-4 | 36818 | 40210 |
|  | 6 | (screen$ or test$).ti,ab,kf. | 4579752 | 5308252 |
|  | 7 | Mandatory Testing/ or Mass Screening/ | 116777 | 123953 |
|  | 8 | (detect$ or predict$ or identif$ or diagnos$ or test$).ti,ab,kf. | 11674637 | 13587882 |
|  | 9 | biomarkers/ or genetic testing/ | 390938 | 445806 |
|  | 10 | (chorionic villus sampling or hplc or high-performance liquid chromatography or MPV or MPH or amniocentesis).ti,ab,kf. | 224987 | 246304 |
|  | 11 | or/6-10 | 12181083 | 14158135 |
| **Study Design** | 12 | Economics/ or exp "Fees and Charges"/ or exp Budgets/ | 69474 | 70272 |
|  | 13 | exp Models, Economic/ or exp Cost-Benefit Analysis/ or exp "Costs and Cost Analysis"/ | 270432 | 289908 |
|  | 14 | Economics, Nursing/ or exp Economics, Medical/ or Economics, Pharmaceutical/ or exp Economics, Dental/ or exp Economics, Hospital/ | 50478 | 51271 |
|  | 15 | Markov Chains/ or Monte Carlo Method/ or Decision Theory/ | 46114 | 50281 |
|  | 16 | (cost$ adj2 (effective$ or utilit$ or benefit$ or minimi$ or consequence$)).ti,ab,kf. | 204768 | 258584 |
|  | 17 | ((economic$ or pharmacoeconomic$ or cost$ or price$ or pricing$ or expenditure$ or financ$) adj2 (evaluat$ or model$ or analys?s or outcome$)).ti,ab,kf. | 92661 | 111537 |
|  | 18 | (value adj2 (money or monetary)).ti,ab,kf. | 3012 | 3550 |
|  | 19 | (economic model$ or markov or monte carlo).ti,ab,kf. | 86507 | 101089 |
|  | 20 | (decision$ adj2 (tree or analys?s or model$)).ti,ab,kf. | 33564 | 49743 |
|  | 21 | exp Value of Life/ or Quality-Adjusted Life Years/ | 21181 | 24195 |
|  | 22 | (quality adjusted life year$ or quality-adjusted life year$ or qaly$ or disability adjusted life year$ or disability-adjusted life year$ or daly$ or life year$ gained or life year$ equivalent$ or incremental cost effective$ or icer or qald$ or qale$ or qtime$).ti,ab,kf. | 30235 | 39323 |
|  | 23 | or/12-22 | 626510 | 729256 |
| **Exclusion** | 24 | exp animals/ not exp humans/ | 5124207 | 5396676 |
|  | 25 | (comment or editorial or case reports or historical article).pt. | 4126507 | 4437132 |
|  | 26 | (case stud$ or case report$).ti. | 378625 | 466293 |
|  | 27 | or/24-26 | 9243943 | 9840492 |
| **Combination** | 28 | 5 and 11 and 23 | 371 | 462 |
|  | 29 | 28 not 27 | 364 | 454 |
| **Time limit** | 30 | limit 29 to yr="2023-current" | N/A | 95 |

**Databases:** Original: Ovid MEDLINE(R) and Epub Ahead of Print, In-Process & Other Non-Indexed Citations and Daily 1946 to 25 May, 2023. Update: Ovid MEDLINE(R) and Epub Ahead of Print, In-Process & Other Non-Indexed Citations and Daily 1946 to 19 November, 2025.

Supplementary Table 2. Search terms for Embase (searched via Ovid SP)

|  |  |  | **Original** | **Update** |
| --- | --- | --- | --- | --- |
|  |  |  | **Date searched: 29th May 2023**  **Records retrieved: 1,386** | **Date searched: 19th November 2025**  **Records retrieved: 626** |
| **Term group** | # | Search terms | Results | Results |
| **Thalassaemia Screening** | 1 | exp thalassemia/ | 38680 | 43868 |
|  | 2 | (thalass?emia or lepore or hydrops fetalis).ti,ab,kf. | 37068 | 41849 |
|  | 3 | ((Mediterranean or erythroblastic or cooley$) adj an?emi$).ti,kf. | 154 | 172 |
|  | 4 | (h?emoglobin adj6 (disorder$ or defect$ or disease$ or trait$)).ti,ab,kf. | 9389 | 11145 |
|  | 5 | or/1-4 | 51806 | 59208 |
|  | 6 | (screen$ or test$).ti,ab,kf. | 6276033 | 7383571 |
|  | 7 | Mandatory Testing/ or Mass Screening/ | 62134 | 67735 |
|  | 8 | (detect$ or predict$ or identif$ or diagnos$ or test$).ti,ab,kf. | 15401557 | 17948186 |
|  | 9 | biomarkers/ or genetic testing/ | 466880 | 765799 |
|  | 10 | (chorionic villus sampling or hplc or high-performance liquid chromatography or MPV or MPH or amniocentesis).ti,ab,kf. | 305112 | 331742 |
|  | 11 | or/6-10 | 15992969 | 18676302 |
| **Study Design** | 12 | economics/ or exp health economics/ or budget/ | 1177807 | 1339913 |
|  | 13 | exp economic model/ or exp economic evaluation/ | 355355 | 408429 |
|  | 14 | exp pharmacoeconomics/ or economic aspect/ | 348884 | 412104 |
|  | 15 | markov chain/ or monte carlo method/ or exp decision theory/ | 61394 | 74558 |
|  | 16 | (cost$ adj2 (effective$ or utilit$ or benefit$ or minimi$ or consequence$)).ti,ab,kf. | 282934 | 353704 |
|  | 17 | ((economic$ or pharmacoeconomic$ or cost$ or price$ or pricing$ or expenditure$ or financ$) adj2 (evaluat$ or model$ or analys?s or outcome$)).ti,ab,kf. | 142528 | 170352 |
|  | 18 | (value adj2 (money or monetary)).ti,ab,kf. | 4105 | 4759 |
|  | 19 | (economic model$ or markov or monte carlo).ti,ab,kf. | 100783 | 117621 |
|  | 20 | (decision$ adj2 (tree or analys?s or model$)).ti,ab,kf. | 46843 | 64917 |
|  | 21 | exp socioeconomics/ or quality adjusted life year/ | 1327091 | 1812413 |
|  | 22 | (quality adjusted life year$ or quality-adjusted life year$ or qaly$ or disability adjusted life year$ or disability-adjusted life year$ or daly$ or life year$ gained or life year$ equivalent$ or incremental cost effective$ or icer or qald$ or qale$ or qtime$).ti,ab,kf. | 49223 | 61939 |
|  | 23 | or/12-22 | 2367791 | 3005364 |
| **Exclusion** | 24 | ("conference abstract" or "conference review").pt. | 4784610 | 5727156 |
|  | 25 | limit 24 to yr="1974-2020" | 4150796 | 4871211 |
|  | 26 | exp animal/ not exp human/ | 5180633 | 5874525 |
|  | 27 | editorial.pt. | 776634 | 852699 |
|  | 28 | editorial/ or case report/ | 3645379 | 4001860 |
|  | 29 | (case stud$ or case report$).ti. | 465526 | 553828 |
|  | 30 | or/25-29 | 12451494 | 14102835 |
| **Combination** | 31 | 5 and 11 and 23 | 1865 | 2705 |
|  | 32 | 31 not 30 | 1386 | 1973 |
| **Time limit** | 33 | limit 32 to yr="2023-current" | N/A | 626 |

**Database:** Original: Embase 1974 to 25 May, 2023. Update: Embase 1974 to 19 November, 2025.

Abbreviations: N/A: not applicable.Supplementary Table 3. Search terms for NHS EED (via York CRD platform)

| **Date searched: 29th May 2023**  **Records retrieved: 19** | | | |
| --- | --- | --- | --- |
| **Term group** | # | Search terms | Results |
| **Thalassaemia Screening** | 1 | MeSH DESCRIPTOR thalassemia EXPLODE ALL TREES | 34 |
|  | 2 | (thalassemia or thalassaemia or lepore or hydrops fetalis) | 67 |
|  | 3 | ((Mediterranean or erythroblastic or cooley*) adj (anemia or anaemia)) | 1 |
|  | 4 | ((hemoglobin or haemoglobin) adj6 (disorder* or defect* or disease* or trait*)) | 20 |
|  | 5 | #1 or #2 or #3 or #4 | 85 |
|  | 6 | (screen* or test*) | 21408 |
|  | 7 | (MeSH DESCRIPTOR Mandatory Testing) | 2 |
|  | 8 | (MeSH DESCRIPTOR Mass Screening) | 2103 |
|  | 9 | (detect* or predict* or identif* or diagnos* or test*) | 35271 |
|  | 10 | (MeSH DESCRIPTOR biomarkers) | 679 |
|  | 11 | (chorionic villus sampling or high-performance liquid chromatography or hplc or MPV or MPH or amniocentesis) | 128 |
|  | 12 | #6 or #7 or #8 or #9 or #10 or #11 | 36553 |
| **Combination** | 13 | #5 and #12 | 50 |
|  | 14 | #13 in NHSEED | 19 |

**Database:** NHS Economic Evaluation Database: Issue 2 of 4, April 2015.

**Abbreviations:** CRD: Centre for Research and Dissemination; EED: Economic Evaluation Database; NHS: National Health Service.

Supplementary Table 4. Search terms for HTAD (via INAHTA)

|  |  |  | **Original** | **Update** |
| --- | --- | --- | --- | --- |
|  |  |  | **Date searched: 29th May 2023**  **Records retrieved: 31** | **Date searched: 19th November 2025**  **Records retrieved: 37** |
| **Term group** | # | Search terms | Results | Results |
| **Thalassaemia Screening** | 1 | "thalassemia"[mhe] | 15 | 27 |
|  | 2 | (thalassaemia or thalassemia or lepore or hydrops fetalis). | 18 | 30 |
|  | 3 | ((Mediterranean or erythroblastic or cooley*) and (anemia or anaemia)). | 1 | 1 |
|  | 4 | ((hemoglobin or haemoglobin) and (disorder* or defect* or disease* or trait*)). | 38 | 44 |
|  | 5 | #1 or #2 or #3 or #4 | 53 | 71 |
|  | 6 | (screen* or test*). | 3090 | 3576 |
|  | 7 | "Mandatory Testing"[mh] or "Mass Screening"[mh] | 697 | 754 |
|  | 8 | (detect* or predict* or identif* or diagnos* or test*). | 6308 | 7361 |
|  | 9 | "biomarkers"[mh] | 186 | 250 |
|  | 10 | (chorionic villus sampling or hplc or high-performance liquid chromatography or MPV or MPH or amniocentesis). | 97 | 123 |
|  | 11 | #6 or #7 or #8 or #9 or #10 | 6779 | 7883 |
| **Combination** | 12 | #5 and #11 | 31 | 37 |

**Database:** Health Technology Assessment Database.

**Abbreviations:** INAHTA: International Network of Agencies for Health Technology Assessment.

Supplementary Table 5. Search terms for CDSR (via Cochrane Library)

|  |  |  | **Original** | **Update** |
| --- | --- | --- | --- | --- |
|  |  |  | **Date searched: 29th May, 2023**  **Records retrieved: 33** | **Date searched: 19th November 2025**  **Records retrieved: 1** |
| **Term group** | # | Search terms | Results | Results |
| **Thalassaemia Screening** | 1 | [mh "thalassemia"] | 503 | 540 |
|  | 2 | (thalassemia or lepore or hydrops fetalis):ti,ab,kw | 1472 | 1679 |
|  | 3 | ((Mediterranean or erythroblastic or cooley*) NEXT (anemia or anaemia)):ti,ab,kw | 15 | 15 |
|  | 4 | (hemoglobin NEAR (disorder* or defect* or disease* or trait*)):ti,ab,kw | 1013 | 1084 |
|  | 5 | #1 or #2 or #3 or #4 | 2410 | 2674 |
|  | 6 | (screen* or test*):ti,ab,kw | 516146 | 615100 |
|  | 7 | [mh ^"mandatory testing"] | 18 | 17 |
|  | 8 | [mh ^"mass screening"] | 4534 | 5244 |
|  | 9 | (detect* or predict* or identif* or diagnos* or test*):ti,ab,kw | 821641 | 980542 |
|  | 10 | [mh ^"biomarkers"] | 18035 | 21585 |
|  | 11 | (chorionic villus sampling or hplc or high-performance liquid chromatography or MPV or MPH or amniocentesis):ti,ab,kw | 10160 | 10354 |
|  | 12 | #6 or #7 or #8 or #9 or #10 or #11 | 860226 | 1026528 |
| **Combination** | 13 | #5 and #12 | 1235 | 1414 |
| **Database limit** | 14 | #13 in Cochrane Database of Systematic Reviews (CDSR)^a^ | 33 | 1 |

**Database:** Original: Cochrane Database of Systematic Reviews: Issue 5 of 12, May 2023. Update: Cochrane Database of Systematic Reviews: Issue 11 of 12, November 2025.

**Footnotes:** [a] Search term for the update was as following: #13 with Cochrane Library publication date Between May 2023 and Nov 2025, in Cochrane Reviews.

**Abbreviations:** CDSR: Cochrane Database of Systematic Reviews.

Supplementary Table 6. Search terms for congress websites

| **Congress** | **Link** | **Search strategy** | **Search terms hits** |
| --- | --- | --- | --- |
| **ASH, December 2021** | [Link](https://ashpublications.org/blood/issue/138/Supplement%201) | The following search string was used within the ‘search within issue’ box:  (Thalassaemia OR thalassemia) AND screen | 15 results, 0 relevant |
| **ASH, December 2022** | [Link](https://ashpublications.org/blood/issue/140/Supplement%201) | The following search string was used within the ‘search within issue’ box:  (Thalassaemia OR thalassemia) AND screen | 21 results, 0 relevant |
| **ASH, December 2023** | [Link](https://ashpublications.org/blood/issue/142/Supplement%201) | The following search string was used within the ‘search within issue’ box:  (Thalassaemia OR thalassemia) AND screen | 64 results, 0 relevant |
| **ASH, December 2024** | [Link](https://ashpublications.org/blood/issue/144/Supplement%201) | The following search string was used within the ‘search within issue’ box:  (Thalassaemia OR thalassemia) AND screen | 71 results, 0 relevant |
| **ASH, December 2025** | [Link](https://ashpublications.org/blood/issue/146/Supplement%201) | The following search string was used within the ‘search within issue’ box:  (Thalassaemia OR thalassemia) AND screen | 71 results, 0 relevant |
| **ASCAT, January 2022** | [Link](https://journals.lww.com/hemasphere/Fulltext/2022/01001/Abstract_Book_for_the_2nd_Sickle_Cell__.1.aspx) | The following terms were searched for sequentially using the ‘Ctrl-F’ search feature of the web page:  Thalassaemia  Thalassemia  Each abstract where the term was mentioned in the title or body of the abstract was then evaluated | 24 results, 0 relevant |
| **ASCAT, October 2022** | [Link](https://journals.lww.com/hemasphere/toc/2023/04001) | The following terms were searched for sequentially using the ‘Ctrl-F’ search feature of the web page:  Thalassaemia  Thalassemia  Each abstract where the term was mentioned in the title or body of the abstract was then evaluated | 19 results, 0 relevant |
| **ASCAT, October 2023** | [Link](https://onlinelibrary.wiley.com/doi/10.1002/hem3.70021) | The following terms were searched for sequentially using the ‘Ctrl-F’ search feature of the web page:  Thalassaemia  Thalassemia  Each abstract where the term was mentioned in the title or body of the abstract was then evaluated | 41 results, 0 relevant |
| **ASCAT, October 2024** | [Link](https://onlinelibrary.wiley.com/doi/10.1002/hem3.70009) | The following terms were searched for sequentially using the ‘Ctrl-F’ search feature of the web page:  Thalassaemia  Thalassemia  Each abstract where the term was mentioned in the title or body of the abstract was then evaluated | 43 results, 0 relevant |
| **ASCAT, October 2025** | [Link](https://onlinelibrary.wiley.com/doi/10.1111/bjh.70197) | The following terms were searched for sequentially using the ‘Ctrl-F’ search feature of the web page:  Thalassaemia  Thalassemia  Each abstract where the term was mentioned in the title or body of the abstract was then evaluated | 44 results, 0 relevant |
| **BSH, April 2022** | [Link](https://onlinelibrary.wiley.com/toc/13652141/2022/197/S1) | The ‘Full Text’ link for the Oral Abstracts, Posters and e-Posters was opened; the following term was searched for using the ‘Ctrl-F’ search feature of the web page:  Thalassaemia  Each abstract where the term was mentioned in the title or body of the abstract was then evaluated | 6 results, 0 relevant |
| **BSH, April 2023** | [Link](https://onlinelibrary.wiley.com/toc/13652141/2023/201/S1) | The ‘Full Text’ link for the Oral Abstracts, Posters and e-Posters was opened; the following term was searched for using the ‘Ctrl‑F’ search feature of the web page:  Thalassaemia  Each abstract where the term was mentioned in the title or body of the abstract was then evaluated | 8 results, 0 relevant |
| **BSH, April 2024** | [Link](https://onlinelibrary.wiley.com/toc/13652141/2024/204/S1) | The ‘Full Text’ link for the Oral Abstracts, Posters and e-Posters was opened; the following term was searched for using the ‘Ctrl‑F’ search feature of the web page:  Thalassaemia  Each abstract where the term was mentioned in the title or body of the abstract was then evaluated | 10 results, 0 relevant |
| **BSH, April 2025** | [Link](https://onlinelibrary.wiley.com/toc/13652141/2025/206/S1) | The ‘Full Text’ link for the Oral Abstracts, Posters and e-Posters was opened; the following term was searched for using the ‘Ctrl‑F’ search feature of the web page:  Thalassaemia  Each abstract where the term was mentioned in the title or body of the abstract was then evaluated | 12 results, 0 relevant |
| **ISPOR, May 2022** | [Link](https://www.ispor.org/heor-resources/presentations-database/search) | The search fields on the linked page were completed as follows:  Disease/Disorder: ALL  Topic: ALL  Conference: 2022-05, ISPOR 2022, Washington  Authors: Leave blank  Keyword: thalassaemia OR thalassemia | 4 results, 0 relevant |
| **ISPOR, May 2023** | [Link](https://www.ispor.org/heor-resources/presentations-database/search) | The search fields on the linked page were completed as follows:  Disease/Disorder: ALL  Topic: ALL  Conference: 2023-05, ISPOR 2023, Boston  Authors: Leave blank  Keyword: thalassaemia OR thalassemia | 12 results, 0 relevant |
| **ISPOR Europe, November 2021** | [Link](https://www.ispor.org/heor-resources/presentations-database/search) | The search fields on the linked page were completed as follows:  Disease/Disorder: ALL  Topic: ALL  Conference: 2021-11, ISPOR Europe 2021, Copenhagen, Denmark  Authors: Leave blank  Keyword: thalassaemia OR thalassemia | 3 results, 0 relevant |
| **ISPOR Europe, November 2022** | [Link](https://www.ispor.org/heor-resources/presentations-database/search) | The search fields on the linked page were completed as follows:  Disease/Disorder: ALL  Topic: ALL  Conference: 2022-11, ISPOR Europe 2022, Vienna, Austria  Authors: Leave blank  Keyword: thalassaemia OR thalassemia | 7 results, 0 relevant |

**Abbreviations:** ASCAT: Annual Sickle Cell & Thalassemia Conference; ASH: American Society of Haematology; BSH: British Society for Haematology; ISPOR: International Society for Pharmacoeconomics and Outcomes Research.

Supplementary Table 7. Search terms for economic websites

|  |  | **Search Strategy** |  | **Search Terms Hits** |  |
| --- | --- | --- | --- | --- | --- |
| **Website** | **Link** | **Original** | **Update** | **Original** | **Update** |
| **The CEA Registry, managed by Tufts Medical Center** | [Link](https://cear.tuftsmedicalcenter.org/) | On the linked page, ‘Methods’ and ‘Advanced’ were selected. ‘Thalassaemia’ was entered as the first keyword, after which ‘+OR’ was clicked and ‘thalassemia’ entered as the second keyword.  All results were cross referenced against the title/abstract review tracker. For any articles that were not captured in the title/abstract review, the abstract was screened against the title/abstract eligibility flowchart. Any abstracts deemed relevant were noted.  The above was repeated with ‘Ratios’ selected and then with ‘Utility Weights’ selected.  This strategy was then repeated for each subsequent search term in the list. | On the linked page, the ‘Advanced’ tab was selected. ‘Thalassaemia’ was entered as the keyword, and ‘Article’ selected as Result Type.  All results were cross referenced against the title/abstract review tracker used for the original review and update. For any articles that were not captured in the title/abstract review, the abstract was screened against the title/abstract eligibility flowchart. Any abstracts deemed relevant were noted.  The above was repeated with ‘Ratio’ selected and then with ‘Utility’ selected.  This strategy was then repeated for ‘thalassemia’. | ‘Methods’ search: 16 results, 0 unique and relevant  ‘Ratios’ search: 20 results, 0 unique and relevant  ‘Utility Weights’ search: 48 results, 0 unique and relevant | ‘Article’ search: 21 results, 0 unique and relevant  ‘Ratio’ search: 23 results, 0 unique and relevant  ‘Utility’ search: 47 results, 0 unique and relevant |

**Footnotes:** In cases where studies that had previously been identified and included through database searches were re-identified in supplementary searches, the number of unique and relevant results have been reported.
**Abbreviations:** CEA: cost-effectiveness analysis.

Supplementary Table 8. Search terms for validation searches

|  |  |  |  | **Search terms hits** | |
| --- | --- | --- | --- | --- | --- |
| **Source** | **Link** | **Search terms** | **Hits Reviewed** | **Original** | **Update** |
| **Google Scholar** | [Link](https://scholar.google.com/) | Cost-effectiveness AND thalassemia AND screening | First 60 | 26 results, 1 unique and relevant | 16 results, 3 unique results, 1 unique and relevant |
|  |  | Cost analysis AND thalassemia AND screening | First 60 | 2 results, 0 relevant | 2 unique results, 0 relevant |
|  |  | Economic AND thalassemia AND screening | First 60 | 3 results, 0 relevant | 0 unique results |
|  |  | Budget AND thalassemia AND screening | First 60 | 3 results, 0 relevant | 0 unique results |
|  |  | Evaluation AND thalassemia AND screening | First 60 | 2 results, 0 relevant | 0 unique results |

**Footnotes:** In cases where studies that had previously been identified and included through database searches were re-identified in supplementary searches, the number of unique and relevant results have been reported.

Supplementary Table 9. Eligibility criteria for the economic evaluations in the economic SLR

| **PICOS Framework** | **Inclusion Criteria** | **Exclusion Criteria** |
| --- | --- | --- |
| **P (Population)** | - Pregnant women, healthy or otherwise - Children aged 2 years or younger, healthy or otherwise - Low- and middle-income countries or high-income countries | Animal subjects |
| **I (Intervention)** | Studies reporting screening programmes for thalassaemia using any available testing strategy, including but not limited to:   - Antenatal screening (e.g. chorionic villus sampling, amniocentesis) - Blood tests (e.g. mean corpuscular haemoglobin, mean corpuscular volume, osmotic fragility test, dichlorophenolindophenol test) - Structural haemoglobin tests (e.g. high-performance liquid chromatography, isoelectric focusing) | - Any other intervention, including treatment of thalassaemia - Studies only reporting screening programmes exclusively for any other haemoglobinopathy, including sickle cell disease, or other disease |
| **C (Comparators)** | Any | None |
| **O (Outcomes)** | Studies reporting outcomes of economic evaluations, including but not limited to:   - Incremental cost-effectiveness ratio - Quality-adjusted life year - Disability-adjusted life year - Life year equivalent/life years gained - Total costs - Cost per clinical outcome - Incremental costs | Studies not presenting relevant outcomes |
| **S (Study design)** | Economic evaluations, including:   - Cost-utility analysis - Cost-consequence analysis - Cost-effectiveness analysis - Cost-benefit analysis - Cost-minimisation analysis - Budget impact analysis | - Any study design other than an economic evaluation, e.g. case studies, editorials - Cost-of-illness analysis |
| **Language** | Title and abstract and/or full text published in English | Title and abstract and/or full text published in any other language |
| **Publication type** | - Congress abstracts published in or after 2021 - Original research studies - Health technology assessments | - Any other publication type, including studies not reporting any original research - Case studies/reports |

Supplementary Table 10. Economic evaluations excluded in the economic SLR (May 2023)

| **#** | **Study name** | **Reference** | **Reason for exclusion** |
| --- | --- | --- | --- |
| **1** | Ahmadnezhad 2012 | Ahmadnezhad, E; Sepehrvand, N; Jahani, FF et al. Evaluation and cost analysis of national health policy of thalassaemia screening in west-azerbaijan province of iran. International Journal of Preventive Medicine. 2012;3(10):687-692. 23112894. | Incorrect population |
| **2** | Avram 2022 | Avram, CM; Caughey, AB; Norton, ME et al. Cost-Effectiveness of Exome Sequencing versus Targeted Gene Panels for Prenatal Diagnosis of Fetal Effusions and Non-Immune Hydrops Fetalis. Am J Obstet Gynecol MFM. 2022;4(6):100724. 35995366. | Incorrect evaluation |
| **3** | Avram 2022 | Avram, CM; Caughey, AB; Norton, ME et al. Cost-Effectiveness of Exome Sequencing versus Targeted Gene Panels for Prenatal Diagnosis of Non-Immune Hydrops Fetalis. American Journal of Obstetrics and Gynecology. 2022;226(1 Supplement):S176-S177. 2016043608. | Incorrect evaluation |
| **4** | Busnelli 2022 | Busnelli, A; Ciani, O; Tarricone, R et al. Implementing preconception expanded carrier screening in a universal healthcare system: insights from a cost-effectiveness analysis. Human Reproduction. 2022;37(Supplement 1):i534. 638854136. | Incorrect population |
| **5** | Cernat 2021 | Cernat, A; Hayeems, RZ; Ungar, WJ. Cascade health service use in family members following genetic testing in children: a scoping literature review. European Journal of Human Genetics. 2021;29(11):1601-1610. | Incorrect study design |
| **6** | Cronin 1998 | Cronin, EK; Normand, C; Henthorn, JS et al. Costing model for neonatal screening and diagnosis of haemoglobinopathies. Arch Dis Child Fetal Neonatal Ed. 1998;79(3):F161-167. 10194984. | Incorrect evaluation |
| **7** | Dormandy 2010 | Dormandy, E; Bryan, S; Gulliford, MC et al. Antenatal screening for haemoglobinopathies in primary care: a cohort study and cluster randomised trial to inform a simulation model. The Screening for Haemoglobinopathies in First Trimester (SHIFT) trial. Health Technol Assess. 2010;14(20):1-160. 20416236. | Incorrect evaluation |
| **8** | Eleftheriou 2022 | Eleftheriou, A; Antoniou, E; Darba, J et al. Estimating the Cost of Thalassemia Care across the World: A Thalassemia International Federation Model. Hemoglobin. 2022;46(6):308-311. 36847683. | Incorrect study design |
| **9** | Esmaeilzadeh 2021 | Esmaeilzadeh, F; Ahmadi, B; Vahedi, S et al. Major Thalassemia, Screening or Treatment: An Economic Evaluation Study in Iran. International Journal of Health Policy & Management. 2021;03:03. 33619933. | Incorrect population |
| **10** | Gessner 1996 | Gessner, BD; Teutsch, SM; Shaffer, PA. A cost-effectiveness evaluation of new-born hemoglobinopathy screening from the perspective of state health care systems. Early Human Development. 1996;45(3):257-275. 21996000829. | Incorrect evaluation |
| **11** | Griffiths 1982 | Griffiths, KD; Raine, DN; Mann, JR. Neonatal screening for sickle haemoglobinopathies in Birmingham. Br Med J (Clin Res Ed). 1982;284(6320):933-935. 6802355. | Incorrect evaluation |
| **12** | Hashempour 2021 | Hashempour, R; Raei, B; Safaei Lari, M et al. QALY league table of Iran: a practical method for better resource allocation. Cost Effectiveness and Resource Allocation. 2021;19(1) (no pagination). 2010175997. | Incorrect study design |
| **13** | Institute of Health 2016 | Institute of Health, E. Newborn blood spot screening for galactosemia, tyrosiemia type I, homocystinuria, sickle cell anemia, sickle cell/beta-thalassemia, sickle cell/hemoglobin C disease and severe combined immunodeficiency. Canada: Institute of Health Economics (IHE); 2016. | Incorrect evaluation |
| **14** | Kim 2021 | Kim, E; Raimundo, K; Marcum, ZA et al. ED2 Elements of Value for Gene Therapy: A Systematic Review. Value in Health. 2021;24(Supplement 1):S6. 2012834407. | Incorrect study design |
| **15** | Le Gales 1989 | Le Gales, C; Moatti, JP. Assessment of screening strategies for haemoglobinopathies in Provence-Alpes-Cote-d'Azur: a comparison of cost-effectiveness and multicriteria analysis. [French]. Journal d'Economie Medicale. 1989;7(2):85-101. 19190770. | Incorrect study design |
| **16** | Milev 2022 | Milev, S; Sardesai, A; Sunil Raj, S et al. POSC143 Comparison of Model Structures Used in NICE and ICER Cost Effectiveness Evaluations. Value in Health. 2022;25(1 Supplement):S115. 2016529068. | Incorrect study design |
| **17** | Nagi 2022 | Nagi, MA; Dewi, PEN; Thavorncharoensap, M et al. A Systematic Review on Economic Evaluation Studies of Diagnostic and Therapeutic Interventions in the Middle East and North Africa. Applied Health Economics and Health Policy. 2022;20(3):315-335. 2014510664. | Incorrect study design |
| **18** | Ostrowsky 1985 | Ostrowsky, JT; Lippman, A; Scriver, CR. Cost-benefit analysis of a thalassemia disease prevention program. American Journal of Public Health. 1985;75(7):732-736. 3923848. | Incorrect population |
| **19** | Phelan 1999 | Phelan, L; Bain, BJ; Roper, D et al. An analysis of relative costs and potential benefits of different policies for antenatal screening for beta thalassaemia trait and variant haemoglobins. Journal of Clinical Pathology. 1999;52(9):697-700. 10655995. | No relevant outcomes |
| **20** | Ratanasiri 2006 | Ratanasiri, T; Charoenthong, C; Komwilaisak, R et al. Prenatal prevention for severe thalassemia disease at Srinagarind Hospital. Journal of the Medical Association of Thailand. 2006;89 Suppl 4:S87-93. 17725145. | Incorrect evaluation |
| **21** | Riku 2021 | Riku, S; Hedriana, H; Carozza, JA et al. Reflex single-gene non-invasive prenatal testing significantly increases the cost-effectiveness of carrier screening. medRxiv. 2021;29. 2012993619. | No relevant outcomes |
| **22** | Riku 2022 | Riku, S; Hedriana, H; Carozza, JA et al. Reflex single-gene non-invasive prenatal testing is associated with markedly better detection of fetuses affected with single-gene recessive disorders at lower cost. Journal of Medical Economics. 2022;25(1):403-411. 35289246. | No relevant outcomes |
| **23** | Scriver 1984 | Scriver, CR; Bardanis, M; Cartier, L. beta-Thalassemia disease prevention: Genetic medicine applied. American Journal of Human Genetics. 1984;36(5):1024-1038. 15205734. | Incorrect population |
| **24** | Sen 2023 | Sen, S; Srivastava, H; Sen, G. Selective screening or universal screening for s-Thalassemia trait - Which is more cost-effective? - A pilot study of a hospital in India. Journal of Obstetrics and Gynaecology Research. 2023;49(Supplement 1):14. 641086710. | Incorrect population |
| **25** | Shah 2022 | Shah, K; Kini, P; Jain, A et al. Prenatal diagnosis of thalassemia: A cost-effective step towards eradication. Pediatric Hematology Oncology Journal. 2022;7(4 Supplement):S9. 2021327993. | Incorrect evaluation |

Supplementary Table 11. Economic evaluations excluded in the economic SLR (December 2025)

| **#** | **Study name** | **Reference** | **Reason for exclusion** |
| --- | --- | --- | --- |
| **1** | Ontario Health 2023 | Ontario Health. Carrier screening programs for cystic fibrosis, fragile x syndrome, hemoglobinopathies and thalassemia, and spinal muscular atrophy: a health technology assessment. Ontario Health Technology Assessment Series. 2023; 23(4):1–398. 37637488 | Duplicate |
| **2** | Abd Rahim 2024 | Abd Rahim, AS; Abd Rahim, NEL; Jaafar, S. A systematic review on thalassaemia screening and birth reduction initiatives: cost to success. The Medical Journal of Malaysia. 2024;79(3):348–359. 38817070 | Incorrect study design |
| **3** | Institute of Health Economics 2016 | Institute of Health Economics. Newborn blood spot screening for galactosemia, tyrosinemia type I, homocystinuria, sickle cell anemia, sickle cell/beta-thalassemia, sickle cell/hemoglobin c disease and severe combined immunodeficiency: costs and cost analysis. Edmonton, Alberta, Canada: Institute of Health Economics (IHE); March 2016. 29708694 | Incorrect evaluation |
| **4** | Jena 2025 | Jena, RK; Sethy, S; Dash, PK et al. Hemoglobin variant analysis and its comparison between conventional high-performance liquid chromatography using whole blood versus dried blood spot: high-performance liquid chromatography. Indian Journal of Public Health. 2025;69(3):280–285. 41045528 | Incorrect study design |
| **5** | Jomoui 2024 | Jomoui, W; Saknava, K; Prechatrammaruch, K et al. Retrospective study and implementation of a low-cost LAMP-turbidimetric assay for screening α0-thalassemia (SEA deletion): preventing and controlling Hb Bart's hydrops fetalis syndrome in Thailand. PeerJ. 2024;12:e17054. 38436007 | Incorrect evaluation |
| **6** | Malaysian Health Technology Assessment 2002 | Malaysian Health Technology Assessment Unit. Maternal screening for foetal abnormality. Malaysia; 2002. | Incorrect study design |
| **7** | Mashon 2025 | Mashon, RS; Mandrelle, K; Kakkar, N. Experience of antenatal thalassemia screening and prenatal diagnosis from a tertiary care teaching hospital in Punjab. Indian Journal of Hematology and Blood Transfusion. 2025;41(1):96–101. 39917488 | Incorrect study design |
| **8** | Milverton 2023 | Milverton, J; Vogan, A; Newton, S et al. MSAC Application no. 1737: Newborn bloodspot screening for sickle cell disease and beta thalassaemia. Australia; 2023. | No relevant outcomes |
| **9** | Musallam 2024 | Musallam, KM; Viprakasit, V; Lombard, L et al. Systematic review and evidence gap assessment of the clinical, quality of life, and economic burden of alpha-thalassemia. EJHaem. 2024;5(3):541–547. 38895066 | Incorrect study design |
| **10** | Ontario Health 2023 | Ontario Health. Carrier screening programs for cystic fibrosis, fragile x syndrome, hemoglobinopathies and thalassemia, and spinal muscular atrophy: a health technology assessment. Ontario Health Technology Assessment Series. 2023; 23(4):1–398. 37637488 | Incorrect evaluation |
| **11** | Sampagar 2023 | Sampagar, A; Patil, NG; Mahantashetti, NS et al. Feasibility and cost analysis of antenatal screening and diagnosis of hemoglobinopathies: a prospective study. Journal of Perinatal and Neonatal Care. 2023;24(1):26–33. | Incorrect study design |
| **12** | Shanthi 2023 | Shanthi, S; Beula, D; Rajendran, A et al. Antenatal screening for haemoglobinopathies among the tribal population in the state of Tamil Nadu, India. Hemasphere. 2023;7:5. | Incorrect study design |
| **13** | Turcotte 2023 | Turcotte, C; Brunet, J; Lalancette-Hébert, M. Pertinence d’ajouter les variantes HbS/E, HbE/E et HbE/β-thal aux cibles primaires du test de dépistage néonatal des hémoglobinopathies. Quebec, Canada; 2023. | Full text not in English |
| **14** | Xi 2023 | Xi, H; Liu, Q; Xie, D et al. Cost-effectiveness analysis of different screening modes for thalassemia in Hunan Province. Chinese Journal of Perinatal Medicine. 2023;26(6):468–475. | Full text not in English |
| **15** | Gupta 2025 | Gupta, R; Radhakrishnan, N; Shankar, R et al. Comparative analysis of the cost of thalassemia screening vs treatment in different healthcare sectors in delhi national capital region. Indian Journal of Public Health Research and Development. 2025;16(2):254–260. | Incorrect evaluation |
| **16** | Bolghasemi 2006 | Abolghasemi, H; Eshghi, P; Rahiminejhad, S et al. Evaluation and cost-effectiveness analysis of prevention program of major thalassemia in Sistan-Balouchestan and Fars provinces. Hakim Research Journal. 2006;8(4):8–14. | Full text not in English |
| **17** | Mittal 2025 | Mittal, AK; Shekhawat, DS; Patel, M et al. Cost-effectiveness analysis of prenatal testing and outcomes for sickle cell disease and thalassemia in India. Preprint; available at Research Square. | Full text not in English |
| **18** | Abd Rahim 2025 | Abd Rahim, AS; Mohamed, DI; Zakaria, Z et al. Cost-effectiveness of multi-sample national thalassemia screening programme vs. single-sample reflex DNA thalassemia screening in Malaysian schools: a comparative study in Sabah and Sarawak. Journal of Hospital Management and Health Policy. 2025;9:39. | Incorrect population |
| **19** | Laoarayawat 2020 | Laoarayawat, T; Bamrungsawad, N; Dilokthornsakul, P et al. Cost-effectiveness analysis of prenatal screening program for thalassemia between semi-accelerated screening step and current program. Ramathibodi Medical Journal 2020;43(1):13–23. | Full text not in English |
| **20** | Mutar 2020 | Mutar, MT; Hameed, MM. Thalassemia prevention program in Iraq: cost-effectiveness and applicability assessment. Baghdad Medical Journal of Students. 2020;1(1):1–9. | Incorrect study design |

Supplementary Table 12. Quality assessments of economic evaluations using items 1–9 of the CASP Economic Evaluation Checklist[1]

| **Study** | **Was a well-defined question posed?** | **Was a comprehensive description of the competing alternatives given?** | **Does the paper provide evidence that the programme would be effective?** | **Were the effects of the intervention identified, measured and valued appropriately?** | **Were all important and relevant resources required, and health outcome costs for each alternative identified, measured in appropriate units and valued credibly?** | **Were costs and consequences adjusted for different times at which they occurred (discounting)?** | **What were the results of the evaluation?** | **Was an incremental analysis of the consequences and cost of alternatives performed?** | **Was an adequate sensitivity analysis performed?** |
| --- | --- | --- | --- | --- | --- | --- | --- | --- | --- |
| **Amarasinghe 2022[2]** | Y | Y | Unable to tell | Y | Y | N | Y | Y | N |
| **Bryan 2011[3]** | Y | Y | Y | Y | Y | Y | Y | Y | Y |
| **Cronin 2000[4]** | N | Y | Y | Y | N | Y | Y | Y | Y |
| **Ginsberg 1998[5]** | Y | Unable to tell | Y | Y | Y | Y | Y | Y | Y |
| **Koren 2014[6]** | Y | Y | Y | Y | Y | N | Y | N | N |
| **Leung 2004[7]** | Y | Unable to tell | Y | Unable to tell | Unable to tell | N | Y | N | N |
| **Malasai 2025a[8]** | Y | Y | Y | Y | Y | Y | Y | Y | Y |
| **Malasai 2025b[9]** | Y | Y | Y | Y | Y | Y | Y | Y | Y |
| **Wiwanitkit 2006[10]** | Y | N | N | N | N | N | Y | N | N |
| **Yang 2016[11]** | Y | Y | Y | Y | Unable to tell | N | Y | Y | N |

**Abbreviations:** N, no; Y, yes.

Supplementary Table 13. Quality assessments of economic evaluations using Q10–Q12 of the CASP Economic Evaluation Checklist[1]

| **Study** | **Is the programme likely to be equally effective in your context or setting?** | **Reasoning** | **Are the costs translatable to your setting?** | **Reasoning** | **Is it worth doing in your setting?** | **Reasoning** |
| --- | --- | --- | --- | --- | --- | --- |
| **Amarasinghe 2022[2]** | Unable to tell | Legal barriers may impact the feasibility of established a screening programme in pregnant women, as was discussed in this study located in Sri Lanka | Y | Sri Lanka is also an LMIC | Unable to tell | It is unclear if Laos and other LMICs may have similar legal barriers to such screening programmes as were present in Sri Lanka |
| **Bryan  2011[3]** | Unable to tell | Demographic and context-specific differences, although the target population of pregnant women is the same | N | England is a HIC and the publication is outdated, so not particularly relevant for LMIC contexts today | N | Many LMICs do not have existing screening programs and that policy option was not considered in this analysis. The publication only compared between different screening strategies |
| **Cronin 2000[4]** | Unable to tell | Demographic and context-specific differences, although the target population of pregnant women is the same | N | England is a HIC; data are low quality data from 30 years ago, so not particularly relevant for LMIC contexts today | Y | The evaluation compares relevant interventions (screening versus no action) |
| **Ginsberg 1998[5]** | Unable to tell | Study is set in a high-income, non-APAC country, meaning the population and social settings likely differ | Unable to tell | Israel is a HIC; study was conducted >20 years ago, so costs are likely outdated | Unable to tell | This is dependent on present screening programmes available and the feasibility of this study's screening programme in Laos and other LMICs |
| **Koren 2014[6]** | Unable to tell | Study set in an HIC, non-APAC country, meaning the population and social settings likely differ | Unable to tell | Israel is a HIC; authors also give the example of Thailand, stating that expenses to treat β-thalassaemia in Thailand are significantly lower than in the Western world | Unable to tell | This is dependent on present screening programmes available and the feasibility of this study's screening programme in Laos and other LMICs |
| **Leung 2004[7]** | Unable to tell | The authors discussed several factors that may affect the cost-effectiveness of the programme, such as the proportion of women presenting themselves for screening after 24 weeks of gestation | Unable to tell | Hong Kong is a HIC, making it difficult to determine how well this evaluation may translate to LMICs | Unable to tell | The authors discussed several factors that affect the feasibility of the screening programme including staff's awareness for the need to screen at-risk ethnic groups, cut off points for MCV abnormality to determine an at-risk mother and prenatal booking practice |
| **Malasai 2025a[8]** | Unable to tell | Thailand seems to have a universal screening programme in place, whereas Laos does not – this means the findings may be a bit less relevant. However, these findings are worth considering in other LMICs given that adding genetic screening has a better cost-benefit ratio and also a bigger budget impact; this is likely to be the case in Laos and other LMICs | Yes | Thailand is an LMIC | No | Many LMICs do not have existing screening programmes, making it difficult to additionally introduce genetic testing as standard practice |
| **Malasai 2025b[9]** | Unable to tell | It is unclear whether Laos would have the capacity to perform screening for both partners | Yes | Thailand is an LMIC | Yes | If there is capacity, a programme screening couples instead of just pregnant women would be worthwhile given these results in a similar setting |
| **Wiwanitkit 2006[10]** | Y | HbE is also prevalent in Laos and many other LMICs | Y | Costs presented in THB (51 THB) translate to about 30000 LAK, which is not expensive | Y | The programme seems to be feasible for implementation in other LMICs |
| **Yang 2016[11]** | Y | Ultrasound is currently available in Laos and may also be available in other LMICs | Y | The cost to do ultrasound in Laos is even 5 times cheaper than in China which suggests ultrasounds may be applicable to other LMIC contexts | Y | The non-invasive programme is similar to that currently used in Laos and may therefore also be used in other LMICs |

**Abbreviations:** APAC: Asia-Pacific; HbE: haemoglobin E; HIC: high-income country; LAK: Lao kip; LMIC: low- and middle-income country; MCV: mean corpuscular volume; N: no; THB: Thai baht; Y: yes.

# REFERENCES

1. Critical Appraisal Skills Programme. CASP Economic Evaluation Checklist 2018 [Available from: Available: https://casp-uk.net/checklists-archive/casp-econonimic-evaluation-checklist-fillable.pdf.

2. Amarasinghe N, Amarasena A, Thabrew A, et al. Redesigning New Policy Options for Thalassemia Prevention in Sri Lanka. Thalassemia Reports. 2022;12:135–42.

3. Bryan S, Dormandy E, Roberts T, et al. Screening for sickle cell and thalassaemia in primary care: a cost-effectiveness study. Br J Gen Pract. 2011;61:e620–7.

4. Cronin EK, Normand C, Henthorn JS, et al. Organisation and cost-effectiveness of antenatal haemoglobinopathy screening and follow up in a community-based programme. Bjog. 2000;107:486–91.

5. Ginsberg G, Tulchinsky T, Filon D, et al. Cost-benefit analysis of a national thalassaemia prevention programme in Israel. J Med Screen. 1998;5:120–6.

6. Koren A, Profeta L, Zalman L, et al. Prevention of β Thalassemia in Northern Israel - a Cost-Benefit Analysis. Mediterr J Hematol Infect Dis. 2014;6:e2014012.

7. Leung KY, Lee CP, Tang MH, et al. Cost-effectiveness of prenatal screening for thalassaemia in Hong Kong. Prenat Diagn. 2004;24:899–907.

8. Malasai K, Jittikoon J, Udomsinprasert W, et al. Cost-Benefit Analysis of Genetic Testing as a Prenatal Diagnostic Tool for Thalassemia: A Single-Center Study From Central Thailand. Clinicoecon Outcomes Res. 2025a;17:33-43.

9. Malasai K, Jittikoon J, Talungchit P, et al. Cost-Effectiveness Analysis of Different Prenatal Screening Strategies for the Prevention of Severe Thalassemia in Thailand. Clinicoecon Outcomes Res. 2025b;17:835-48.

10. Wiwanitkit V. A cost utility analysis of the right method for screening hemoglobin E among Thai pregnant women. Arch Gynecol Obstet. 2006;274:88–90.

11. Yang Y, Li DZ, He P. A Program on Noninvasive Prenatal Diagnosis of α-Thalassemia in Mainland China: A Cost-Benefit Analysis. Hemoglobin. 2016;40:247–9.
