# Supplementary material for: Systematic review of economic evaluations in thalassaemia screening programmes globally: developing guidance for low- and middle-income (LMIC) settings
Source: BMJ Open. 2026 May 6;16(5):e108768. doi: 10.1136/bmjopen-2025-108768 (PMC13150896; doi:10.1136/bmjopen-2025-108768)
Supplement: online supplemental file 1 [file bmjopen-16-5-s002.pdf]

AUTHOR REFLEXIVITY STATEMENT

How does this study address local research and policy priorities?

This study was initiated by members of the Mahidol Oxford Tropical Medicine Research Unit (MORU), a research collaboration between Mahidol University (Thailand), Oxford University (UK) and the Wellcome Trust (UK), in response to a Global Health Call for Projects put forth to non-profits by Costello Medical in 2023. The research question was posed by MORU as part of their initiative to establish a Unit for Health Evidence and Policy, which aims to strengthen evidence-based decision making in Laos and introduce health technology assessment into the policy process. Thalassaemia screening has been identified as a priority topic for an HTA study in the country, owing to thalassemia being a neglected and high priority public health issue.

The aim of the systematic literature review was therefore to support a future HTA study in Laos, by making it easier for a local non-health economics experts and/or trainee researchers to conduct an economic evaluation on this topic, ultimately supporting a reduction in disease incidence and cost burden. As such, although the study was conducted collaboratively with Costello Medical, the subject matter reflects the local research and policy demands.

How were local researchers involved in study design?

The local research team, namely KP, EAA, MM and CP, were integral to the study's conception and design. Furthermore, the protocol was collaboratively developed by all the authors. While KM, VL and AM of Costello Medical were responsible for providing technical expertise and training, the local researchers were pivotal in initially proposing the study's topic and concept for a systematic literature review.

How has funding been used to support the local research team?

This study was conducted free-of-charge on a pro bono basis by Costello Medical. Although MORU is funded by the Wellcome Trust, Costello Medical's contribution allowed this specific study to proceed without incurring additional costs for the local research team.

How are research staff who conducted data collection acknowledged?

All individuals involved in data collection are recognised as authors on the publication.

Do all members of the research partnership have access to study data?

Yes, all members involved in the research partnership have access to study data.

How was data used to develop analytical skills within the partnership?

All authors were involved in the extraction and/or interpretation of data from the studies included in the systematic literature review thereby helping them develop and refine their analytical skills.

How have research partners collaborated in interpreting study data?

KM, KP, VL, AM and CP were responsible for extracting data from the studies included in the systematic literature review, with MM and EEA having access to the extraction grid. All members of the research partnership engaged in discussions about data interpretation throughout the manuscript development process.

How were research partners supported to develop writing skills?

Costello Medical provided writing support free-of-charge on a pro bono basis for this study; however, all research partners were invited to review and refine the writing. Additionally, all team members were provided training on report writing.

How will research products be shared to address local needs?

This systematic literature review will be published as open access. In addition, the local research team will be able to use the outputs of the research (including the extraction grid, report and manuscript) to facilitate local initiatives around evidence-based decision making.

Economic evaluations of thalassaemia screening in LMICs Author Reflexivity Statement

How is the leadership, contribution and ownership of this work by LMIC researchers recognised within the authorship?

The team involved in the project are entirely represented within the authorship list, which has been constructed in line with ICMJE criteria. Also, the last author on the study (CP) is based in the global south. The data is held by both the non-local and local research team, and the researchers involved are able to use it for further analysis, teaching or other non-commercial purposes.

How have early career researchers across the partnership been included within the authorship team?

This partnership did not include any early career researchers.

How has gender balance been addressed within the authorship?

Three authors are male (MM, KP, CP) and four authors female (KM, VL, AM, EA).

How has the project contributed to training of LMIC researchers?

Local researchers were provided training related to economic evaluations, economic systematic literature reviews, data extractions and report writing.

How has the project contributed to improvements in local infrastructure?

This project has not directly contributed to improvements in local infrastructure.

What safeguarding procedures were used to protect local study participants and researchers?

There was no primary data collection as part of this project, therefore this question is not directly applicable.
