# Supplementary material for: Systematic review of economic evaluations in thalassaemia screening programmes globally: developing guidance for low- and middle-income (LMIC) settings
Source: BMJ Open. 2026 May 6;16(5):e108768. doi: 10.1136/bmjopen-2025-108768 (PMC13150896; doi:10.1136/bmjopen-2025-108768)
Supplement: Supplementary file 3 [file bmjopen-2025-108768.reviewer_comments.pdf]

PEER REVIEW HISTORY

BMJ Open publishes all reviews undertaken for accepted manuscripts. Reviewers are asked to complete a checklist review form and are provided with free text boxes to elaborate on their assessment. These free text comments are reproduced below.

This paper was submitted to another journal from BMJ but declined for publication following peer review. The authors addressed the reviewers' comments and submitted the revised paper to BMJ Open. The paper was subsequently accepted for publication at BMJ Open.

ARTICLE DETAILS

Title (Provisional)

Systematic review of economic evaluations in thalassaemia screening programmes globally: developing guidance for low- and middle-income (LMIC) settings

Authors

Massey, Katherine; Phommasone, Koukeo; Mehta, Aditi; Lee, Vivienne Qi Yan; Ashley, Elizabeth A; Mayxay, Mayfong; Painter, Chris

---

VERSION 1 - REVIEW

**Reviewer**	**1**
**Name**	**Reviewer 1**
**Affiliation**	
**Date**	**21-May-2025**
**COI**	**No competing interest**

Comments not published pending reviewer's consent

**Reviewer**	**2**
**Name**	**Saygın Avşar, Tuba**
**Affiliation**	**University College London, Health Economics Unit**
**Date**	**21-May-2025**
**COI**	**No competing interest**

I'd like to thank the authors for their work on this important topic. I think the paper needs substantial improvement to be published in a high-profile journal. I am confused about the setting of the paper. The title and objective state that the focus is LMICs. However, the inclusion and exclusion criteria does not specify settings, and studies from England were also included. This needs to be clarified. Additionally, the paper intends to explore the methods followed in economic evaluations, but methodological details of the economic evaluations are not provided in detail. Also, the authors should go beyond merely

reporting what was done and discuss the appropriateness of the methods followed. For example, what is the appropriate time horizon for an economic evaluation of thalassaemia screening programmes? Were the modelling approaches appropriate? I am also a bit concerned that the literature search did not cover any LMIC-sensitive database, e.g. Global Index Medicus (WHO).
There are a few error codes in the text – these should be corrected.
I've provided some specific comments below.
Title: Given the study included HICs as well as LMICs, I think the title should not include LMICs.
What does this study add? - Does the author refer to cost-utility analysis here? I think cost-benefit analysis can also be considered an economic evaluation.
Methods:
What does dual-reviewer process mean? Were all records screened in duplicate at all stages (title & abstract, full-text)?
How was "economic evaluation" defined? Were partial economic evaluations (e.g. cost-minimisation) included? Some readers might not be familiar with the health economics terminology, so I'd suggest including a definition with citation to credible sources.
Is Table 1 really needed? I think it occupies so much space unnecessarily. Could these be provided as a list with bullet points?
Table 1 - Could abbreviations be written in full in this table for consistency? (e.g. ICER, QALYs, etc.). Not clear if budget impact analysis was included or excluded.
Data Extraction – I'd argue that quality and risk of bias are not necessarily the same thing.
Why was "Critical Appraisal Skills Programme (CASP) Economic Evaluation Checklist" chosen? This tool is not sufficient for model-based economic evaluations.
Results:
I would expect the results section to include sub-sections focussing on health outcomes and resource use and costs. These sub-sections could detail which parameters were considered in the economic evaluations assessed and how. I would also expect a section on quality assessment where the quality and robustness are discussed in depth.
Table 2 – NR (not reported) should be included in the abbreviations list.
Table 3 – should include more details about the economic evaluations, e.g. type (trial-based or model-based), currency and price year, etc. The authors already attempted some price conversions. For consistency, either all costs reported should be reported in the same currency and price year or everything should be reported as in the original paper.
The results section needs to be strengthened with more details on the methodology used in the economic evaluations. For example, what does this sentence mean "one adopted both a healthcare sector and societal perspective"? How was the societal perspective defined in that study? Was it only productivity costs that were included in addition to the health system costs? Similarly, what was the difference between the "healthcare payer" and "healthcare sector" perspectives?
Details of the sensitivity analyses could e provided. Were the base-case findings supported by the sensitivity analyses? It is stated that "Costs associated with lifetime care were considered in four studies". How was this done? Were the methods consistent with the internationally accepted practice?
In this section, I would also expect a comparison of studies from HICs and LMICs. For instance, was the study using the societal perspective from a HIC?
Discussion:
The discussion mainly focusses on two points: the appropriate economic evaluation type (cost benefit analysis) and perspective (societal perspective). How about the other methodological considerations, such as time horizon?
I find these two sentences a bit confusing "this SLR only captured evaluations of antenatal screening programmes, with no evaluations of post-natal screening programmes identified. However, in the future, when gene therapy is more accessible, post-natal screening may be an important alternative to consider." Could it be made explicit that no study identified in this review evaluated post-natal screening programmes? Could some additional information be provided about post-natal screening programmes with some references to key studies?

VERSION 1 - AUTHOR RESPONSE

Reviewer 2:

I'd like to thank the authors for their work on this important topic. I think the paper needs substantial improvement to be published in a high-profile journal.

Response: We thank the reviewer for their review of the paper and comments.

I am confused about the setting of the paper. The title and objective state that the focus is LMICs. However, the inclusion and exclusion criteria does not specify settings, and studies from England were also included. This needs to be clarified.

Response: We thank the reviewer for their comment and have accordingly clarified the setting throughout the paper, including within the Title, Abstract and Introduction:

Title, page 1, lines 1–2:

> "Systematic review of economic evaluations in thalassaemia screening programmes globally: developing guidance for low- and middle-income (LMIC) settings"

Abstract, on page 2, lines 8–9:

> "Studies conducted in any geographic setting were eligible for inclusion. Findings were interpreted within the context of LMIC's, given the high prevalence and resource limitations in these settings."

Introduction, on page 4, lines 1–3:

> "The objective of this study was to conduct a systematic literature review (SLR) to investigate how economic evaluations of thalassaemia screening programmes have been carried out globally, in order to identify best practices for performing future evaluations in LMIC contexts."

Additionally, the paper intends to explore the methods followed in economic evaluations, but methodological details of the economic evaluations are not provided in detail. Also, the authors should go beyond merely reporting what was done and discuss the appropriateness of the methods followed. For example, what is the appropriate time horizon for an economic evaluation of thalassaemia screening programmes? Were the modelling approaches appropriate

Response: The provided methodological details have been expanded for all included economic evaluations, to provide more detail on the approaches used, perspectives taken, time horizons, discounting rates, sensitivity analyses undertaken and costs considered, in the Results, on pages 8–9.

Due to the methodological variation observed across the included studies, drawing firm recommendations with regards to these components is challenging. Nonetheless, we have added a point to the Discussion, on page 15, lines 20–30, to clarify this and provide suggestions:

> "Due to the methodological variation observed across the included studies, drawing firm recommendations with regards to these components remains challenging. Nonetheless, sensitivity analyses examining different perspectives (e.g. societal versus healthcare sector) and treatment costs could provide insight into the robustness of the conclusions. Discount rates should align with local or national standards to ensure accurate reflection of the time value of money. Employing a lifetime time horizon that accurately accounts for average life expectancy of a person living with thalassaemia in the geography of interest would also help ensure a comprehensive evaluation is undertaken. Separately, the variance in the heterogeneity in prevalence of thalassaemia across countries could be taken into account.[9-11] For example, scenario analyses could be conducted for regions or provinces as the cost-effectiveness of screening may vary accordingly. These types of scenario analyses could help LMICs target particularly burdened areas, thereby helping maximise the impact of the screening programmes and ensure that limited resources are used most effectively."

I am also a bit concerned that the literature search did not cover any LMIC-sensitive database, e.g. Global Index Medicus (WHO).

Response: We thank the reviewer for their comment. Given that studies conducted in any geographic location were eligible for inclusion, we did not search any LMIC-sensitive databases. However, we acknowledge that this is a methodological limitation, and have accordingly noted this in the Discussion, on page 15, lines 41–44:

> "Moreover, only publications written in English were included in the review, and no non-English databases, like Global Index Medicus, were searched; as such, relevant literature published in the languages of countries which experience significant burden of thalassaemia would not have been identified."

There are a few error codes in the text – these should be corrected.

Response: We have corrected these error codes.

Title: Given the study included HICs as well as LMICs, I think the title should not include LMICs.

Response: We thank the reviewer for their comment, and have updated the title to clarify the setting and objective of the work:

> Systematic review of economic evaluations in thalassaemia screening programmes globally: developing guidance for low- and middle-income (LMIC) settings

What does this study add? - Does the author refer to cost-utility analysis here? I think cost-benefit analysis can also be considered an economic evaluation.

Response: We thank the reviewer for their comment. *BMJ Open* does not require this section, and as such this comment is no longer relevant.

Methods:

What does dual-reviewer process mean? Were all records screened in duplicate at all stages (title & abstract, full-text)?

Response: We have added more detail about the review process to the Methods, on page 4, lines 37–41:

> "Potentially eligible studies were screened based on title and abstract by two authors independently (KP, VL, AM, CP). If necessary, any disagreements were resolved by discussion until a consensus was met, or with the assistance of a third independent reviewer (KM). Final selection of studies was based on full-text reviews, which were undertaken by two authors independently (KP, VL, AM, CP); as before, any disagreements were resolved by discussion or, if necessary, with the assistance of a third independent reviewer (KM)."

How was "economic evaluation" defined? Were partial economic evaluations (e.g. cost-minimisation) included? Some readers might not be familiar with the health economics terminology, so I'd suggest including a definition with citation to credible sources

Response: We thank the reviewer for their comment and suggestion. We have clarified the types of economic evaluations that were eligible for inclusion, and provided a reference containing definitions of these for readers to review in the Methods, on page 4, lines 30–32:

> "Economic evaluations[21] (cost-utility analyses [CUAs], cost-consequence analyses, cost-effectiveness analyses [CEA], cost-benefit analyses [CBA], cost-minimisation analyses and budget impact analyses)…"

The reference we have used is as following:

> Turner HC, Archer RA, Downey LE, et al. An Introduction to the Main Types of Economic Evaluations Used for Informing Priority Setting and Resource Allocation in Healthcare: Key Features, Uses, and Limitations. *Front Public Health* 2021; **9**: 722927.

Is Table 1 really needed? I think it occupies so much space unnecessarily. Could these be provided as a list with bullet points? Could abbreviations be written in full in this table for consistency? (e.g. ICER, QALYs, etc.).

Response: Table 1 has been shifted to the Supplementary Material, such that it is now Supplementary Table 9. The content of Table 1 has been summarised in the Methods accordingly, on page 4, lines 28–36:

> "Studies were eligible for inclusion if they met the eligibility criteria (Supplementary Table 9). In brief, included studies were those reporting the outcomes of economic evaluations of screening programmes for thalassaemia for pregnant women or children aged two years or younger. Economic evaluations21 (cost-utility analyses [CUAs], cost-consequence analyses, cost-effectiveness analyses [CEA], cost-benefit analyses [CBA], cost-minimisation analyses and budget impact analyses) conducted in any country and reporting outcomes including (but not limited to) incremental cost-effectiveness ratios (ICERs), quality-adjusted life years (QALYs) or disability-adjusted life years (DALYs), life years gained and incremental costs were eligible for inclusion. The exclusion criteria included, but was not limited to, studies in animal subjects, studies not presenting relevant economic outcomes, and studies reporting screening programmes exclusively for any other disease."

Further, as requested, abbreviations have been written out in full throughout the table.

Not clear if budget impact analysis was included or excluded.

Response: We can confirm that budget impact analyses were eligible for inclusion. This has been noted in both Supplementary Table 9, and within the Methods, on page 4, lines 30–32:

> "Economic evaluations[21] (cost-utility analyses [CUAs], cost-consequence analyses, cost-effectiveness analyses [CEA], cost-benefit analyses [CBA], cost-minimisation analyses and budget impact analyses)…"

Data Extraction – I'd argue that quality and risk of bias are not necessarily the same thing.

Response: We thank the reviewer for their comment, and have updated the text to clarify that the Critical Appraisal Skills Programme (CASP) Economic Evaluation Checklist was used to assess quality only.

Why was "Critical Appraisal Skills Programme (CASP) Economic Evaluation Checklist" chosen? This tool is not sufficient for model-based economic evaluations.

Response: While alternative tools were considered for this SLR, including Drummond's Checklist for the Critical Appraisal of Economic Evaluations, it was ultimately decided to proceed with the CASP Economic Evaluation Checklist due to the study types that were identified in the SLR (i.e. non-model-based evaluations). We have clarified this within the Methods, on page 5, lines 5–9:

> "The quality of all included articles was assessed using the Critical Appraisal Skills Programme (CASP) Economic Evaluation Checklist;[22] quality assessment was performed by a single author for each included study, and then independently verified by a second author (KP, VL, AM, CP). Any discrepancies identified by the second author were arbitrated by a third author (KM). The CASP Economic Evaluation Checklist provides a systematic approach to the critical appraisal of economic evaluations of a broad range of methodologies."

Results:

I would expect the results section to include sub-sections focussing on health outcomes and resource use and costs. These sub-sections could detail which parameters were considered in the economic evaluations assessed and how. I would also expect a section on quality assessment where the quality and robustness are discussed in depth.

Response: We thank the reviewer for their suggestion, and have introduced sub-sections within the Results for parameters of the economic evaluations, including approaches, perspectives, time horizons, discounting rates, sensitivity analyses, costs and assumptions. Furthermore, we have added a specific section for findings related to quality assessment.

Table 2 – NR (not reported) should be included in the abbreviations list.

Response: We can confirm that this abbreviation is included in the abbreviations list.

Table 3 – should include more details about the economic evaluations, e.g. type (trial-based or model-based), currency and price year, etc. The authors already attempted some price conversions. For consistency, either all costs reported should be reported in the same currency and price year or everything should be reported as in the original paper.

Response: We thank the reviewer for their comment, and have updated the table to report the type of economic evaluation and cost year. Further, we now report all results as they are reported in the original papers (i.e. we have removed all price conversions).

Results: The results section needs to be strengthened with more details on the methodology used in the economic evaluations. For example, what does this sentence mean "one adopted both a healthcare sector and societal perspective"? How was the societal perspective defined in that study? Was it only productivity costs that were included in addition to the health system costs? Similarly, what was the difference between the "healthcare payer" and "healthcare sector" perspectives? Details of the sensitivity analyses could be provided. Were the base-case findings supported by the sensitivity analyses? It is stated that "Costs associated with lifetime care were considered in four studies". How was this done? Were the methods consistent with the internationally accepted practice?

Response: We have expanded the Results section to discuss how the different parameters were considered within each included study.

In this section, I would also expect a comparison of studies from HICs and LMICs. For instance, was the study using the societal perspective from a HIC?

Response: Due to the death of literature identified and the variety of modelling approaches observed, such comparisons are challenging to make and, if performed, would lack robustness. Nonetheless, we have noted instances where some parameters (e.g. sensitivity analyses) were only reported in studies based in high-income countries.

Discussion:

The discussion mainly focusses on two points: the appropriate economic evaluation type (cost benefit analysis) and perspective (societal perspective). How about the other methodological considerations, such as time horizon?

Response: Due to the varied approaches taken across all included studies, coupled with the fact that very few studies reported parameters like time horizon and discount rates, firm recommendations were difficult to draw. We have updated the Discussion to note this and provided some broader recommendations on how such methodological components could be considered for inclusion, on page 15, lines 18–30:

> "Despite the pragmatic nature of the recommended CBA, robustness and credibility of results should still be maximised as far as possible. For example, the evaluation should include a clear study objective, description and rationale for the competing alternative, perform sensitivity analyses, incorporate discounting, and specify a lifetime time horizon. Due to the methodological variation observed across the included studies, drawing firm recommendations with regards to these components remains challenging. Nonetheless, sensitivity analyses examining different perspectives (e.g. societal versus healthcare sector) and treatment costs could provide insight into the robustness of the conclusions. Discount rates should align with local or national standards to ensure accurate reflection of the time value of money. Employing a lifetime time horizon that accurately accounts for average life expectancy of a person living with thalassaemia in the geography of interest would also help ensure a comprehensive evaluation is undertaken. Separately, the variance in the heterogeneity in prevalence of thalassaemia across countries could be taken into account.9-11 For example, scenario analyses could be conducted for regions or provinces as the cost-effectiveness of screening may vary accordingly. These types of scenario analyses could help LMICs target particularly burdened areas, thereby helping maximise the impact of the screening programmes and ensure that limited resources are used most effectively."

I find these two sentences a bit confusing "this SLR only captured evaluations of antenatal screening programmes, with no evaluations of post-natal screening programmes identified. However, in the future, when gene therapy is more accessible, post-natal screening may be an important alternative to consider." Could it be made explicit that no study identified in this review evaluated post-natal screening programmes? Could some additional information be provided about post-natal screening programmes with some references to key studies?

Response: We thank the reviewer for their comment. Economic evaluations of post-natal screening programmes were eligible for inclusion, however none were identified. We have re-phrased these sentences to clarify this within the Discussion, on page 15, lines 44–47:

> "Lastly, it is important to highlight that this SLR only captured evaluations of antenatal screening programmes; no study identified in this review evaluated post-natal screening programmes. However, should potentially curative gene therapy become more accessible in LMICs, economic evaluations of post-natal screening programmes may become more prevalent, and therefore may be an important alternative to consider."

VERSION 2 - REVIEW

**Reviewer**	**2**
**Name**	**Saygın Avşar, Tuba**
**Affiliation**	**University College London, Health Economics Unit**
**Date**	**30-Oct-2025**
**COI**	**None**

I would like to thank the authors for their work. The manuscript has been improved substantially. I have no further comments.

**Reviewer**	**4**
**Name**	**Kar, Anita**
**Affiliation**	**Birth Defects and Childhood Disability Research Centre**
**Date**	**31-Aug-2025**
**COI**	**None**

The authors report a systematic literature review of economic evaluation of thalassemia screening programmes, with the goal of identifying how future economic evaluations should be conducted in LMICs. In this revised version of the manuscript, the authors have addressed the reviewer's comments, which has strengthened the overall quality of the article. The article is very well written, the conclusions are meaningful and the article will be an important contribution for policy-makers implementing screening for haemoglobinopathies.

**Reviewer**	**5**

Name Garg, Akanksha

Affiliation Zydus Hospitals Ahmedabad, Hematology and BMT

Date 23-Sep-2025

COI None

The authors have presented a well written and balanced systematic review. However, I have the following comments-

1. Introduction can be shortened and points discussed here can be mentioned in the discussion.

VERSION 2 - AUTHOR RESPONSE

Reviewer 2:

I would like to thank the authors for their work. The manuscript has been improved substantially. I have no further comments.

Response: We thank the reviewer for their review of the paper and feedback.

Reviewer 4:

The authors report a systematic literature review of economic evaluation of thalassemia screening programmes, with the goal of identifying how future economic evaluations should be conducted in LMICs. In this revised version of the manuscript, the authors have addressed the reviewer's comments, which has strengthened the overall quality of the article. The article is very well written, the conclusions are meaningful and the article will be an important contribution for policy-makers implementing screening for haemoglobinopathies.

Response: We thank the reviewer for their review of the paper and feedback.

Reviewer 5:

The authors have presented a well written and balanced systematic review. However, I have the following comments-

1. Introduction can be shortened and points discussed here can be mentioned in the discussion.

Response: We thank the reviewer for their review of the paper and comments. We have revised the Introduction to be more concise, in line with this suggestion. Specifically, we have condensed the background information on thalassaemia.
